# Myeloid-derived suppressor cells as immunosuppressive regulators and therapeutic targets in cancer

**DOI:** 10.1038/s41392-021-00670-9

**Published:** 2021-10-07

**Authors:** Kai Li, Houhui Shi, Benxia Zhang, Xuejin Ou, Qizhi Ma, Yue Chen, Pei Shu, Dan Li, Yongsheng Wang

**Affiliations:** 1 Department of Thoracic Oncology, State Key Laboratory of Biotherapy and Cancer Center, West China Hospital, Sichuan University and Collaborative Innovation Center, 610041 Chengdu, China; 2 Department of Gynecology and Obstetrics, Key Laboratory of Birth Defects and Related Diseases of Women and Children, Ministry of Education, West China Second Hospital, Sichuan University, 610041 Chengdu, China; 3 Institute of Respiratory Health, Frontiers Science Center for Disease-related Molecular Network, and Precision Medicine Center, Precision Medicine Key Laboratory of Sichuan Province, West China Hospital, Sichuan University, 610041 Chengdu, China; 4 Clinical Trial Center, West China Hospital, Sichuan University, 610041 Chengdu, China

**Keywords:** Cancer microenvironment, Tumour immunology

## Abstract

Myeloid-derived suppressor cells (MDSCs) are a heterogenic population of immature myeloid cells with immunosuppressive effects, which undergo massive expansion during tumor progression. These cells not only support immune escape directly but also promote tumor invasion via various non-immunological activities. Besides, this group of cells are proved to impair the efficiency of current antitumor strategies such as chemotherapy, radiotherapy, and immunotherapy. Therefore, MDSCs are considered as potential therapeutic targets for cancer therapy. Treatment strategies targeting MDSCs have shown promising outcomes in both preclinical studies and clinical trials when administrated alone, or in combination with other anticancer therapies. In this review, we shed new light on recent advances in the biological characteristics and immunosuppressive functions of MDSCs. We also hope to propose an overview of current MDSCs-targeting therapies so as to provide new ideas for cancer treatment.

## Introduction

The overall survival of cancer patients has been greatly extended in recent years due to improved healthcare. However, malignant tumors still remain one of the leading causes of deaths around the world, with almost 10 million cancer deaths occurring in 2020.^1^ The great success of immune checkpoint inhibitors in cancer immunotherapy has attracted more and more studies focusing on immune cell populations and signaling pathways with immunosuppressive effects, in order to develop more efficient immunotherapeutic approaches. Myeloid-derived suppressor cells (MDSCs), characterized by their suppressive effects on immune responses, are important motivators to promote tumor immune escape. MDSCs expand massively along with tumor progression, and play critical roles in tumor development, metastasis, and treatment resistance. There has been increasing evidence indicating that MDSCs are one of the fundamental features of malignant tumors, as well as potential therapeutic targets for cancer treatment.^2^

MDSCs originate from hematopoietic stem cells (HSCs) as a result of an altered myelopoiesis. At steady-state, myelopoiesis is a structured process to sustain the stable supply of host myeloid cells. Bone marrow (BM)-derived HSCs give rise to immature myeloid cells (IMCs), which terminally differentiate into monocytes (further differentiating into macrophages and dendritic cells (DCs)) and granulocytes (including neutrophils, basophils, and eosinophils). A variety of pathological conditions such as infection or tissue damage can initiate emergency myelopoiesis to eliminate the potential threats to the host.^3^ In these conditions, myeloid cells rapidly mobilize from the BM and are classically activated in response to pathogenic signals such as toll-like receptor (TLR) ligands, damage-associated molecular patterns (DAMPs), and pathogen-associated molecular patterns (PAMPs), resulting in dramatic increases in phagocytosis, respiratory burst, and upregulation of proinflammatory cytokines.^4^ This transient myelopoiesis terminates upon the elimination of stimulus, and then the homeostasis of myeloid cells is restored. However, some pathological conditions such as chronic inflammation, cancer, and autoimmune disease can lead to an aberrant, sustained myelopoiesis to prevent the host from extensive tissue damage caused by unresolved inflammation.^5–7^ In these conditions, persistent inflammation signals make IMCs deviate from normal differentiation and pathologically activated. Compared to physiologically differentiated myeloid cells, these IMCs have distinct features such as immature phenotypes and morphologies, relatively weak phagocytic activities, as well as anti-inflammatory and immunosuppressive functions, which are now collectively termed as MDSCs.^6^

In recent years, studies on the role of MDSCs in cancer have profoundly expanded our knowledge of tumor pathobiology. MDSCs are characterized by their abilities to suppress immune responses and shield tumor cells from the host immune attack. Besides, they also contribute to tumor progression through various non-immunological mechanisms such as promoting vascularization and pre-metastatic niche formation.^8^ MDSCs expansion has been observed in both cancer patients and tumor-bearing mice, and the frequencies of MDSCs in circulation and tumor site are correlated positively with tumor burden but negatively with antitumoral therapy response and overall survival (OS) in tumor-bearing hosts.^9–11^ In addition, numerous studies have indicated that MDSCs act as a valuable prognostic biomarker for cancer development, as well as a potential target for anticancer therapies.^12,13^ Currently, multiple novel drugs targeting MDSCs have been investigated in preclinical and clinical studies. At the same time, a number of conventional drugs have been reported to be effective in depleting MDSCs and consequently improve the efficacy of cancer immunotherapy.^14^

In this review, we delineated the development and characteristics of MDSCs, as well as their biological roles in tumor progression, and reviewed current MDSCs-targeting approaches. In summary, this review provides an overview of the characteristics and immunosuppressive roles of MDSCs, along with a detailed discussion on MDSCs-targeting therapies in cancer.

## A brief history of MDSCs investigation in cancer

**Fig. 1:**
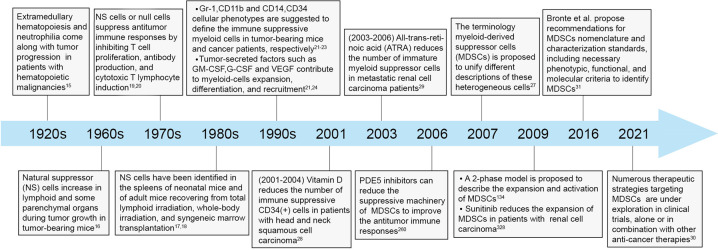
Historical progression in the investigation of MDSCs.

Studies of MDSCs in cancer can be traced back to the early 1900s when Sonnenfeld et al. found that extramedullary hematopoiesis and neutrophilia came along with tumor progression in patients with hematopoietic malignancies.^15^ In the mid-1960s, pathologic leukemoid reaction and increased myeloid cells infiltration were found in A-280 tumor-bearing mice, which were motivated by tumor-derived factors and were positively associated with tumor growth.^16^ In addition, these myeloid cells had also been found in inflammatory and hematopoietic processes, such as the spleens of neonatal mice and the spleens of adult mice receiving total lymphoid irradiation.^17,18^ In the 1970s, these abnormal myeloid cells were identified to have the properties to inhibit antibody production, T cell proliferation, and cytotoxic T lymphocyte (CTL) induction, and were described as natural suppressor (NS) cells, veto cells, or null cells in disparate studies due to the lack of classic membrane markers of T cells, B cells, natural killer cells (NKs), or macrophages.^19,20^ Until the late 1990s, the surface markers Gr-1 and CD11b were identified to define these immune suppressive myeloid cells in tumor-bearing mice.^21^ Also, in cancer patients, these populations of myeloid cells were described based on their expression of CD34 and CD14 as well as their capabilities to suppress T cells.^22,23^ Nevertheless, descriptions of these cells in later investigations were diverse, including myeloid suppressor cells (MSCs), immature myeloid cells, and Gr1^+^ myeloid cells.^24–26^ Until 2007, the MDSCs terminology was proposed to describe these heterogeneous cells, and most investigators accepted this nomenclature since then.^27^ Around that period, studies on the strategies targeting these clusters of cells also made meaningful progress. Notably, in the early 2000s, vitamin D and all trans-retinoic acid (ATRA) administration were demonstrated to induce the differentiation of immature myeloid cells and reduce their immunosuppressive function in patients with head and neck squamous cell carcinoma (HNSCC) and metastatic renal cell carcinoma (mRCC) respectively.^28,29^ After that, new drugs were developed increasingly to target MDSCs, most of which could induce a superior tumor control when added to the existing therapeutic regimens. More importantly, several conventional drugs have shown synergistic effects in cancer patients by effectively depleting MDSCs when combined with traditional antitumor therapies (Fig. 1).^30^ It is foreseeable that MDSCs-targeting therapies will become an important compliment to current cancer treatment strategies in the near future.

## Development and characteristics of MDSCs

Based on the density, morphology, and phenotype, MDSCs fall mainly into two subsets: polymorphonuclear (PMN)-MDSCs and monocytic (M)-MDSCs. PMN-MDSCs were initially termed as granulocytic (G)-MDSCs. Gradually, the term PMN-MDSCs gained more popularity, since it makes this cluster of cells distinguishable from steady-state neutrophils in the aspects of morphology and phenotype (such as having altered buoyancy, less granules, reduced CD16, CD62L, and upregulated CD11b, CD66b).^31^ Additionally, a group of more immature progenitor cells are defined as “early-stage MDSCs” (eMDSCs) with the phenotype of CD11b^+^Gr-1^+^CCR2^+^Sca1^+^CD31^+^ in mouse and CD33^+^HLA-DR^−^ Lin^−^ in human.^31–33^ Besides, a unique population of fibrocystic MDSCs (F-MDSCs) has been described and characterized in human.^34,35^

**Fig. 2:**
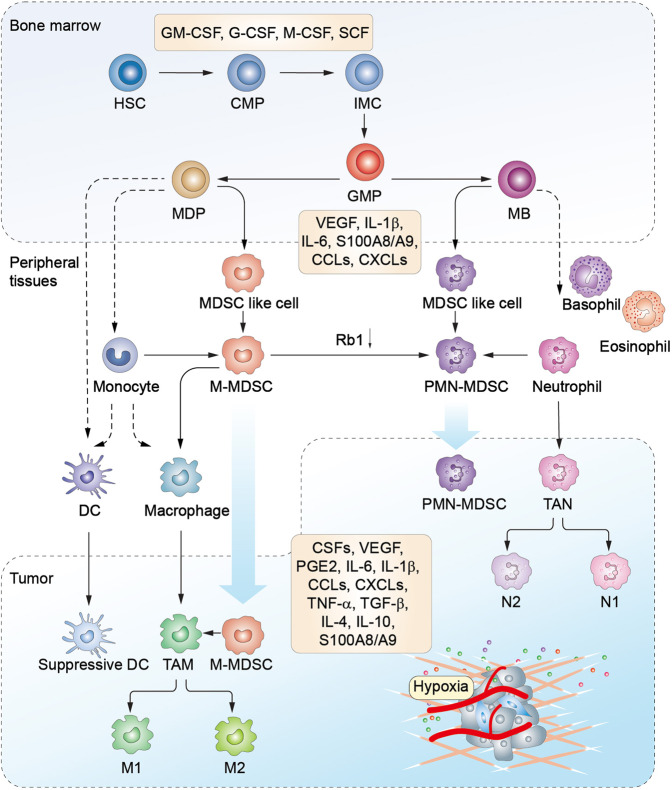
Schematic diagram of MDSCs development, recruitment, and differentiation. In the bone marrow (BM), hematopoietic stem cells (HSCs)-derived common myeloid progenitors (CMPs) give rise to granulocyte-macrophage progenitors (GMPs) expansion. GMPs further differentiate into macrophage/dendritic cell progenitors (MDPs) and myeloblasts (MBs). This myelopoiesis process is controlled by growth factors such as GM-CSF, G-CSF, M-CSF, and SCF, etc. In normal physiological condition, as illustrated with the dotted line, MDPs further increase and are converted to macrophages and dendritic cells (DCs). MBs are further converted to granulocytes including basophils, eosinophils, and neutrophils. Under cancer conditions, lager population of immature myeloid cells (IMCs) are pathologically activated and then differentiate into M-MDSCs and PMN-MDSCs in the presence of tumor-derived factors such as VEGF, IL-6, and IL-1β, etc. In early tumor stages, cells with similar biochemical features as MDSCs do not have suppressive activity, and are reffered as MDSC-like cells. MDSCs may also arise partially from reprogramming of the existing differentiated monocytes and polymorphonuclear cells. M-MDSCs can differentiate into PMN-MDSCs through transcriptional silencing of the retinoblastoma gene (Rb1). MDSCs are recruited into peripheral tissues and tumor microenvironment (TME) under chemotaxis of several factors, such as CCL2, CXCLs, and S100A8/A9, etc. In the TME, M-MDSCs can further differentiate into tumor-associated macrophages (TAMs), and TAMs may acquire M1 or M2 phenotypes. Tumor-associated neutrophils (TANs) can be classified as tumor-inhibitory N1 and tumor-promoting N2 subtypes. M1, type 1 TAM; M2, type 2 TAM; N1, type 1 TAN; N2, type 2 TAN.

**Fig. 3:**
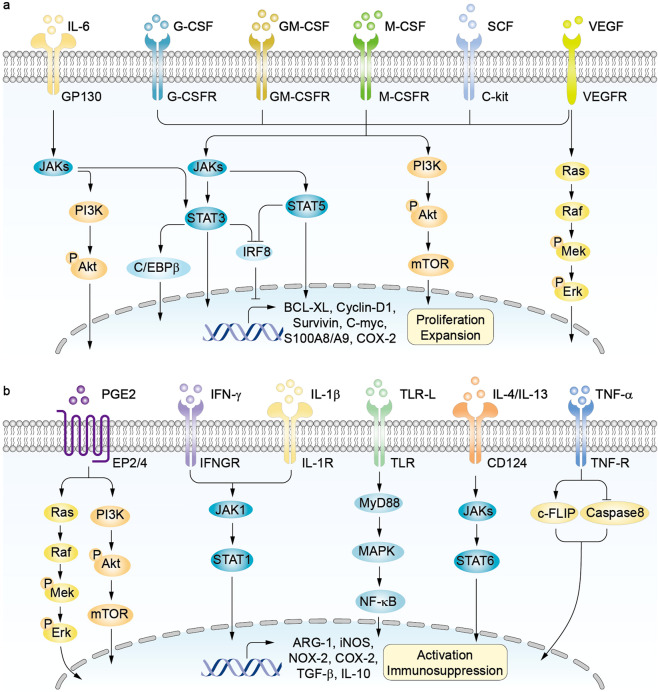
Signaling pathways of MDSCs expansion and activation. **a** CSFs, SCF, IL-6 and VEGF are key promoters of MDSCs proliferation and expansion. The process is regulated by JAKs-STATs, PI3K-Akt, and Mek-Erk signaling pathways, leading to the expression of BCL-XL, Cyclin-D1, and C-myc in MDSCs. MDSCs expansion is inhibited by interferon-related factor 8 (IRF-8). G-CSF and GM-CSF downregulate IRF-8 expression through modulation of STAT3 and STAT5, respectively. **b** Numerous cancer-associated factors mediate the activation and immunoregulatory function of MDSCs. PGE2 activates EP2/4 signaling through Mek-Erk and PI3K-Akt pathways. IFN-γ and IL-1β activate MDSCs through JAK1-STAT1 pathway. Proinflammatory danger signals such as S100A8/A9 proteins and high-mobility grow box-1 (HMGB1) enhance MDSCs trafficking and function by binding to TLRs. IL-4 and IL-13 function through IL-4Rα-dependent STAT6 activation. TNF-α activates TNF-R signaling by upregulating cellular FLICE-inhibitory protein (c-FLIP) expression and by suppressing caspase-8 activity. All these signals can induce the expression of inhibitory factors such as ARG-1, iNOS, NOX-2, COX-2, TGF-β, and IL-10 in MDSCs.

A two-phase model was proposed to describe the development of MDSCs in the context of cancer.^36^ The first expansion phase involves IMCs proliferation in the BM, which is induced by various factors produced by tumors or the BM stroma.^36^ Currently, it has been gradually accepted that during myelopoiesis in the BM, a similar procedure referred to as extramedullary myelopoiesis is initiated in the peripheral organs, such as spleen.^37^ The second activation phase involves the conversion of IMCs to MDSCs in peripheral tissues, which is mainly motivated by pro-inflammatory cytokines derived from tumor-associated stromal cells and activated T cells. This 2-phase model suggests that the accumulation of MDSCs is realizable only when the signals of the two phases are provided simultaneously.^38^ Recently, Karin proposed a four-step event to characterize the development of MDSCs (step I–IV: myelopoiesis, mobilization to the blood, homing to the tumor site, retention at the tumor site) from a migratory viewpoint (Fig. 2).^39^ This model is not contrary to the two-phase model, but adds two additional steps (III, IV) to provide detailed and complementary information associated with the migratory properties of MDSCs. On the other hand, the signal factors initiate all the processes mentioned above through inducing crosstalk between HSCs and tumor tissues. Overall, these signals overlap significantly. It involves growth factors including granulocyte-macrophage colony-stimulating factor (GM-CSF), granulocyte (G)-CSF, macrophage (M)-CSF, stem cell factor (SCF),^40^ and vascular endothelial growth factor (VEGF); cytokines such as interleukin (IL)-4, IL-6, IL-10, IL-1β, interferon (IFN)-γ, tumor necrosis factor (TNF)-α,^41^ transforming growth factor (TGF)-β,^42^ and prostaglandin E2 (PGE2); alarmins like high-mobility grow box-1 (HMGB1)^43^ and S100 calcium-binding protein A8/A9 (S100A8/A9); chemokines such as C–C motif chemokine ligand 2 (CCL2), C–X–C motif chemokine ligand 5 (CXCL5), and CXCL12; enzymes like cyclooxygenase-2 (COX-2) and indoleamine 2,3-dioxygenase (IDO). These signals also include tumor-derived exosomal proteins, RNAs, and microRNAs.^44^ Furthermore, these signals form an interactive communication network under the modulation of transcription factors,^45^ such as signal transducer and activator of transcription (STAT), nuclear factor kappa-B (NF-κB), CCAAT enhancer-binding protein-β (C/EBPβ), and NOTCH (Fig. 3).

### Expansion and recruitment of MDSCs

Several unique mechanisms involved in the development of MDSCs have been proposed and verified in recent years. CSFs (G-CSF, GM-CSF, and M-CSF) were demonstrated to drive emergency hematopoiesis in tumors by upregulating a critical transcription factor, retinoic-acid-related orphan receptor C1 (RORC1).^46^ Additionally, GM-CSF and G-CSF negatively regulate interferon-related factor 8 (IRF-8) through STAT3 and STAT5 signaling pathways to reverse IRF-8-mediated hindering effect on MDSCs formation.^47^ Recently, reduced retinol metabolism and attenuated retinoic acid (RA) signaling were proved to provide a possible driving force that fostered the generation of PMN-MDSCs in colorectal tumors.^48^ On the other hand, infiltration of newly formed MDSCs to tumor sites are recruited by tumor-associated C–C and C–X–C motif chemokines and their receptors.^49^ These chemokines are not unique for specific cancer types and have high redundancy to sustain the constant migration of MDSCs. The CCL2/CCR2 axis is the main pathway implicated in monocytes/M-MDSCs migration,^50^ while neutrophils/PMN-MDSCs are recruited mainly by the CXCLs-CXCR1/2 axis.^51^ Moreover, it was found that the expression of chemokine CCL26 in tumor cells induced the accumulation of CX_3_CR1^+^ MDSCs,^52^ and the CCR5/CCR5 ligand axis also supported the maintenance of MDSCs in the tumor niches.^53^

### Differentiation of MDSCs

At the early stage of cancer, MDSCs are rarely detectable, however, there are a group of cells sharing similar genomic and biochemical characteristics with MDSCs but lacking immunosuppressive activities, which are termed as MDSC-like cells.^31,54^ There is a controversy concerning the origin of MDSCs. Single-cell transcriptomics revealed that MDSCs derived from distinct clusters of neutrophilic and monocytic lineages^55^, while another hypothesis proposed that MDSCs arose partially from reprogramming of classical monocytes and polymorphonuclear cells.^56^ In addition, MDSCs in the periphery and tumor tissues can further differentiate. It was recently reported that a large proportion of M-MDSCs could differentiate into PMN-MDSCs in tumor-bearing mice, and transcriptional silencing of the retinoblastoma gene (Rb1) via epigenetic modifications by histone deacetylase 2 (HDAC-2) mediated this phenotype conversion.^57^ In the periphery, the existence of tumor-derived inflammatory factors promotes the differentiation of M-MDSCs into immunosuppressive macrophages as well as inhibits the functional maturation of DCs. Whereas in the hypoxic TME, M-MDSCs can differentiate into tumor-associated macrophages (TAMs).^58^ A recent study reported that tumor-infiltrating M-MDSCs downregulated STAT3 activity via hypoxia-induced activation of CD45 phosphatase to promote their rapid differentiation into TAMs.^59^ Additionally, tumor-associated neutrophils (TANs) are classified into N1 (tumor-inhibitory) and N2 (tumor-promoting) subtypes based on their functional differences.^60^ It was hypothesized that N2 TANs in the TME were either periphery-recruited PMN-MDSCs or periphery-derived neutrophils, which obtained an N2 phenotype under the stimulation of TGF-β. Based on the N1/N2 classification, N2 neutrophils and PMN-MDSCs appear to be equivalent or the same population essentially.^61^ Therefore, a better definition of PMN-MDSCs is necessary to provide a consensus, especially within the context of the N1/N2 classification.

### Functional regulation of MDSCs

Different mechanisms are involved in the regulation of immunosuppressive functions of MDSCs. It was reported that MDSCs dramatically upregulated TAM RTKs (TYRO3, AXL, MERTK transmembrane receptor tyrosine kinases) and the corresponding ligands to mediate immunosuppression in tumor-bearing mice and metastatic melanoma patients.^62^ A recent study revealed that the long noncoding RNA Pvt1 (lncRNA Pvt1) also significantly regulated the immunosuppressive activities of PMN-MDSCs in tumor-bearing mice.^63^ More importantly, tumor represents a special state of stress, which is thought to greatly contribute to the generation and suppressive function of MDSCs.^64,65^ MDSCs derived from cancer patients and tumor-bearing mice were found to overexpress endoplasmic reticulum (ER) stress markers such as C/EBP homologous protein (CHOP) and spliced X-box binding protein 1 (sXBP1),^58^ furthermore, the ER stress response level was substantially higher at the tumor site than in the peripheral lymphoid organs in tumor-bearing mice.^65^ Interestingly, activation of stress-induced β2-adrenergic receptors (ARs) and expression of reactive oxygen species (ROS)-mediated TIPE2 (TNF-α-induced protein 8-like 2) in MDSCs were both reported to mediate the immunosuppressive functions of MDSCs.^66,67^ In summary, these mediators may be regarded as promising therapeutic targets to reduce MDSCs-mediated immunosuppression in cancer.

### Survival of MDSCs

MDSCs turnover varies with cancer types, with a half-life of a few days. Activated T cells contribute to this rapid turnover of MDSCs since FasL^+^ T cells can induce the apoptosis of Fas^+^ MDSCs.^68^ Additionally, it has been found that MDSCs in tumor-bearing mice had a shorter half-life and lower viability than classical neutrophils and monocytes because of increased apoptosis mediated by the ER stress-induced expression of TRAIL-Rs (TNF-related apoptosis-induced ligand receptors) in MDSCs.^65^ However, MDSCs also have some anti-apoptosis mechanisms. It was demonstrated that the inflammatory environment could increase MDSCs resistance to Fas-FasL signaling-mediated lysis, resulting in a longer half-life of MDSCs in vivo.^69^ MDSCs were also proved to increase BCL-XL (B cell lymphoma XL) expression to deregulate Fas-FasL signaling-induced apoptosis and to escape the elimination by host CTLs.^70^ More importantly, it was found that M-MDSCs required continuous c-FLIP (cellular FLICE-inhibitory protein) expression to prevent cell death, whereas PMN-MDSCs required the anti-apoptotic molecule MCL-1 (myeloid cell leukemia 1) to counter the intrinsic apoptotic pathway.^71^ Besides, TNF was also reported to promote MDSCs survival by upregulating c-FLIP and inhibiting the activity of caspase-8.^72^

### Identification of MDSCs

At present, the same phenotypical characteristics used to identify neutrophils and monocytes are also used for identifying PMN-MDSCs and M-MDSCs, respectively. However, some newly reported methods and molecular markers may help to further distinguish these two pairs of cells.^5,31,73^

One method allowing for the distinguishment between PMN-MDSCs and neutrophils in the peripheral blood is the standard Ficoll gradient centrifugation. In healthy individuals, PMN-MDSCs are rarely detectable in the peripheral blood mononuclear cell (PBMC) fraction. In tumor-bearing hosts, the high-density fraction of neutrophils (HDNs) are classical neutrophils which were previously described as N1-type neutrophils (Nc1), which have antitumor effects such as phagocytosis and antibody-dependent cytotoxicity. The low-density fraction (mononuclear cell fraction) consists of at least two morphologically distinct neutrophil subsets: activated mature neutrophils (circulating N2-type neutrophils, Nc2) and immature PMN-MDSCs,^74^ and both subsets display pro-tumor properties.^75^ Mature Nc2 in the low-density fraction are derived from mature HDNs in a TGF-β-dependent manner. Since low density and immunosuppressive activity are two defining characteristics of PMN-MDSCs as described in numerous studies, both mature Nc2 and immature PMN-MDSCs can be qualified as MDSCs.^74^ Therefore, there is a need for a unified nomenclature of immunosuppressive neutrophils.^76^ Nevertheless, this method has some limitations: some PMN-MDSCs can pass through the low-density gradient and in turn contaminate HDNs, and the results rely heavily on the collection and storage conditions of the blood. On the other hand, there are no established methodologies currently to unequivocally distinguish between PMN-MDSCs and immunosuppressive TANs in tumor tissues.^77^ Notably, LOX-1 (lectin-type oxidized LDL receptor 1) was identified recently in humans to separate PMN-MDSCs from neutrophils without the need of gradient centrifugation.^78^ In cancer patients, LOX-1^+^ immunosuppressive cells with PMN-MDSCs features accounted for 5–15% of neutrophils in the blood and up to 50% of neutrophils in tumor tissues.^79^ However, these cells were practically undetectable in the peripheral blood of healthy individuals.

M-MDSCs and classical monocytes can be discriminated based on MHC-II molecules expression in the peripheral blood of cancer patients. M-MDSCs have the CD11b^+^CD14^+^CD15^–^CD33^+^HLA-DR^–/lo^ phenotype, whereas monocytes are HLA-DR positive.^76^ However, phenotype alone is possibly insufficient to fully distinguish M-MDSCs from monocytes, making the distinguishment between this pair of cells in tumor-bearing mice much more challenging. Fortunately, a recent study on single-cell RNA sequencing in breast cancer confirmed that cell surface receptors CD84 and JAML (junction adhesion molecule like) could be used in combination with CD11b/Gr-1 or CD11b/CD15/CD14 to detect MDSCs in mouse breast cancer model and breast cancer patients, respectively. However, it remains to be determined whether these findings are applicable to other cancers.^55^ Additionally, Khan et al. recently found that, among the cells with e-MDSCs phenotype markers in patients with ovarian cancer, 58% in blood and 36% in ascites were basophils on the basis of cytology and high CD123 expression, while immature cells were rare. This suggests that e-MDSCs phenotype markers need to be re-evaluated to exclude basophils.^80^

Therefore, future studies in terms of genomic, proteomic, molecular, and functional characterizations are wanted to specifically identify MDSCs populations.^81^ Bronte et al. proposed an algorithm including necessary phenotypic, functional, and molecular criteria to identify MDSCs, which provides a unified framework for future MDSCs research.^31^

## MDSCs-mediated tumor-promoting effects

MDSCs utilize multiple mechanisms to dampen antitumor immunity and promote tumor progression. For one thing, MDSCs contribute to the formation of an immunosuppressive milieu which in turn exerts influence on the biology and function of MDSCs. For another, MDSCs also support tumor progression and induce antitumoral therapy resistance in various non-immunological manners.

### MDSCs-mediated suppression on immune responses

The immune defense system, mainly comprising cytotoxic T lymphocytes, NK cells, antigen presenting cells (APCs), and B cells, is indispensable in tumor control and elimination although it is always disrupted by immune inhibitory cells. Notably, in tumor-bearing hosts, MDSCs play a critical role in facilitating tumor immune escape by inhibiting tumoricidal immune cells as well as through acting in league with other inhibitory immune cells.

#### Expression of negative immune checkpoint molecules

Numerous studies have revealed that MDSCs increase PD-L1 expression to induce T-cell anergy through interacting with PD-1 on T cells.^82,83^ Tumor-infiltrating MDSCs always come with higher PD-L1 expression compared with their counterparts in the periphery, indicating their acclimatization in the hypoxic microenvironment.^82,83^ Interestingly, Cassetta et al. reported that in cancer patients, profound PD-L1 expression was restricted to M-MDSCs and e-MDSCs, whereas LOX-1 expression was confined to PMN-MDSCs.^7^ Besides, MDSCs also express cytotoxic T lymphocyte-associated antigen 4 (CTLA-4), although the specific regulating mechanism is unclear. Blocking CTLA-4 has been reported to dampen the accumulation of granulocytic MDSCs and reduce their arginase 1 (ARG1) production in the peripheral blood of patients with metastatic melanoma.^84^

**Fig. 4:**
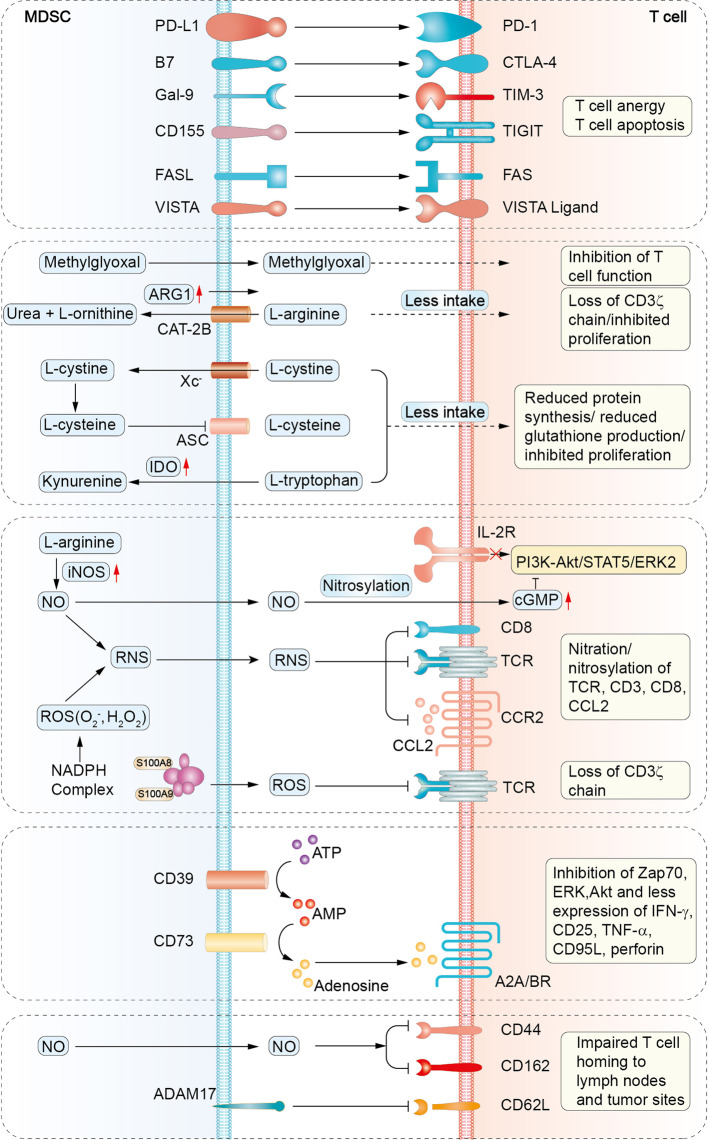
Mechanisms of MDSCs-mediated T cells suppression. MDSCs suppress T cell activity through distinct mechanisms including expression of negative immune checkpoint molecules, depletion of amino acids necessary for T cell response, production of NO, ROS, RNS, generation of adenosines, and impairment of T cell homing to peripheral lymph nodes and tumor sites. TIM-3, T cell immunoglobulin and mucin domain-3; Gal-9, galectin-9; TIGIT, T cell immunoglobulin and ITIM domain; VISTA, V-domain Ig-containing suppressor of T-cell activation; CAT-2B, cationic amino acid transporter 2B; ASC, asc­type amino acid transporter; Xc^–^, cystine–glutamate transporter; IDO, indole-2,3 dioxygenase; iNOS, inducible nitric oxide synthase; NO, nitric oxide; TCR, T cell receptor; Zap70, zeta-associated protein 70; ERK, extracellular-signal-regulated kinase; Akt, protein kinase B; ADAM17, a disintegrin and metalloproteinase domain 17.

Recently, some other immune checkpoint molecules, such as VISTA (V-domain Ig-containing suppressor of T-cell activation), Gal-9 (galectin-9), and CD155, have been reported in MDSCs-mediated immunosuppression (Fig. 4). In the peripheral blood of acute myeloid leukemia (AML) patients, high VISTA expression on MDSCs was positively associated with T cell-expressed PD-1,^85^ while blockade of VISTA was proved to allow the restoration of the protective antitumor response in mouse melanoma models.^86^ Additionally, T cell-expressed TIM-3 (T cell immunoglobulin and mucin domain 3) can interact with Gal-9 on MDSCs to promote MDSCs expansion and suppress T cells responses.^87^ The TIM-3/Gal-9 pathway was demonstrated to be critical in primary and secondary resistance to anti-PD-1 treatment in metastatic non-small cell lung cancer (NSCLC) patients.^88^ Gal-9 has also been reported to promote myeloid lineage-mediated immunosuppression in TME by enhancing the degradation of STING.^89^ Moreover, TIGIT (T cell immunoglobulin and ITIM domain) is an inhibitory regulator expressed on T lymphocytes, and the TIGIT/CD155 pathway is involved in tumor-infiltrating T cell exhaustion.^90,91^ Recent studies have indicated that CD155 expression on MDSCs contributes to MDSCs-mediated T cell inhibition, and targeting the TIGIT/CD155 pathway in vitro with anti-TIGIT antibody significantly abrogated the immunosuppressive activities of MDSCs.^92^ In summary, these researches suggest that immune checkpoint molecules expressed on MDSCs negatively regulate T cells functions.

#### Depletion of amino acids required for T cell response

MDSCs are notorious for deprivation of essential amino acids required for T cells metabolism and function. Large amounts of TME-derived factors, such as HIF-1, TGF-β, IL-4, IL-10, and IFN-γ, can induce the expression of CAT-2B (cationic amino acid transporter) and ARG1 in MDSCs.^93,94^ CAT-2B rapidly transfers extracellular L-arginine into MDSCs, which is subsequently degraded into urea and L-ornithine under the catalysis of ARG1.^94^ Consequently, the deficiency of arginine in the extracellular space can lead to the CD3ζ chain loss and apparent proliferation inhibition of T cells.^95^ In cancer patients, MDSCs were reported to release ARG1 into the extracellular environment, which also resulted in a consumption of extracellular L-arginine and further facilitated T cells inhibition in a similar manner.^96^ Notably, MDSCs have been recently reported to induce T cell suppression through the cell–cell transfer of methylglyoxal into T cells. Methylglyoxal suppressed T cells not only through depleting cytosolic L-arginine but also by rendering L-arginine-containing proteins non-functional through glycation.^97^ In addition, MDSCs can uptake cystine and metabolize it into cysteine. However, due to the lack of neutral amino acid transporter, MDSCs cannot export cysteine back to the extracellular environment, leading to the deprivation of cysteine for T cell activation.^98^ Furthermore, depletion of tryptophan through IDO in MDSCs can induce T cell autophagy, cell cycle arrest, and even cell death.^99^

#### Production of NO, ROS, and RNS

MDSCs secrete a serious of reactive oxygen and nitrogen species to damage T cell function. Upregulated inducible nitric oxide synthase (iNOS) in MDSCs metabolizes L-arginine into nitric oxide (NO) and L-citrulline. NO drives several molecular blockades in T cells, including interference with IL-2R signaling and nitration of T cell receptors (TCRs) specific for the peptides presented by MDSCs.^100,101^ ROS, comprising oxygen radicals (such as superoxide anion, O_2_^−^), hydroxyl radicals, and non-radicals (such as hydrogen peroxide, H_2_O_2_), are generated in high amounts by the NADPH oxidase isoform (NOX-2) in MDSCs. ROS not only play an important role in oxidative stress of MDSCs, but also catalyze the nitration of TCR/CD8 molecules to prevent the TCR/MHC-peptide interactions.^102^ Furthermore, O_2_^−^ combines with NO rapidly to produce reactive nitrogen species (RNS) such as peroxynitrite (ONOO^−^), which can induce the nitration/nitrosylation of TCR/CD8 molecules and further elicit an altered TCR/MHC-peptide recognition.^103,104^ Notably, RNS were reported to induce the posttranslational modification of CCL2, and the reduced affinity of CCL2 to CCR2 consequently inhibited the recruitment of tumor-infiltrating lymphocytes (TILs) to tumor tissues.^105^ However, it does not lead to complete loss of myeloid cells function, because myeloid cells have higher CCR2 expression levels than CD8^+^ T cells.^105^

#### Adenosines and adenosine receptors

The immunosuppressive factor adenosine has also been proved to participate in MDSCs-mediated T cell suppression.^106^ Hypoxic tumor tissues release high amounts of adenosine triphosphates (ATPs) in the extracellular space, which are immediately degraded into adenosines. In this process, CD39 converts ATP to adenosine diphosphate (ADP) and/or adenosine monophosphate (AMP), and CD73 catalyzes the generation of adenosine from AMP.^107^ The accumulated extracellular adenosines activate downstream signaling pathways through adenosine receptors: A2AR, A2BR (both are typically associated with profound immunosuppression), A1R, and A3R. In the TME, these adenosinergic molecules (CD39, CD73, A2AR, and A2BR) are generally expressed by tumor cells as well as stromal and immune cells, forming a positive feedback loop. This feedback produces a constant stream of adenosines, which not only facilitate the development and immunosuppressive capability of MDSCs, but also impair the activities of tumoricidal immune cells, including T cells, DCs and NK cells.^108^ In a mouse melanoma model, Umansky et al. proposed that tumor cells, MDSCs, and regulatory T cells (Tregs) could generate extracellular adenosines in a paracrine manner to inhibit T-cell function.^109^ Moreover, it was reported that a fraction of activated MDSCs from NSCLC patients expressed both CD39 and CD73, which was correlated with cancer progression and chemotherapy resistance.^110^

#### Impairment of T cell trafficking

MDSCs utilize a variety of ways to impair T cells trafficking in tumor-bearing hosts. ADAM17 (a disintegrin and metalloproteinase domain 17) expressed on MDSCs directly cleaves the ectodomain of L-selectin (CD62L) on naive T cells to inhibit them homing to peripheral lymph nodes and tumor sites.^111^ Moreover, downregulation of CD44 and CD162 on T cells by M-MDSCs-derived NO can damage T cells extravasation and tissue infiltration.^112^ In addition, NO was also reported to decrease E-selectin expression on tumor vessels, thereby inhibiting T cells trafficking to tumor tissues.^113^

#### Crosstalk between MDSCs and other immune cells

Apart from T cells, MDSCs also deliver immune inhibition on other tumoricidal immune cells such as NK cells, DCs, and B cells. It was reported that membrane-bound TGF-β1 on MDSCs contributed to suppressing the innate immune function of NK cells in mouse tumor models.^114^ Moreover, M-MDSCs from liver cancer patients were found to cause autologous NK cells anergy in vitro, mainly via the interaction of NKp30 receptor on NK cells with NKp30 ligand on MDSCs.^115^ Additionally, PMN-MDSCs were reported to block antigen cross-presentation of DCs by transferring oxidized lipids from PMN-MDSCs to DCs in tumor-bearing mice.^116^ In melanoma patients, high frequencies of M-MDSCs in the starting culture of monocytes could inhibit DCs maturation and resulted in impaired overall quality of monocytes-derived DC vaccines.^117^ MDSCs also can impair the function of B cells to suppress humoral immune responses. In a lung cancer mouse model, MDSCs inhibited the differentiation and function of B cells by modulating IL-7 and downstream STAT5 signaling.^118^ In a breast cancer mouse model, MDSCs upregulated PD-L1 expression on B cells, and further transformed them into regulatory B cells (Bregs) which had higher inhibitory abilities on T cells.^119^ What’s more, splenic MDSCs from tumor-bearing mice were reported to downregulate the adhesion molecule L-selectin on splenic B cells, resulting in reduced B cells homing to lymph nodes.^120^

On the other hand, MDSCs can incite other immune inhibitory cells such as Tregs and TAMs to facilitate immunosuppression. In mouse tumor models, it was demonstrated that tumor-infiltrating M-MDSCs could produce CCR5 ligands to chemoattract Tregs with high CCR5 expression into tumor tissues.^121^ Furthermore, MDSCs can induce Tregs proliferation through either a direct cell-cell interaction or secretion of soluble factors like IL-10 and TGF-β.^122^ The expression of ARG1, IDO, and CD40 by MDSCs have also been reported to participate in Tregs induction.^123^ Macrophage is another accomplice of MDSCs. The cell–cell interactions between MDSCs and macrophages can elicit a type 2 tumor-promoting immune response, which is mediated by elevated IL-10 production in MDSCs and downregulated IL-12 production in macrophages.^124^ Overall, MDSCs together with other immune suppressive cells build an inhibitory network, crippling the cytotoxic effects on tumor cells.

### Non-immunological functions of MDSCs

Other than the effects on immune responses, MDSCs also contribute to tumor progression via multiple non-immunological mechanisms such as supporting angiogenesis, promoting stemness of tumor cells, facilitating epithelial–mesenchymal transition (EMT) and pre-metastatic niche formation.^8,125^ MDSCs not only exploit abundant VEGFs in the TME but also generate VEGFs themselves, creating a positive feedback loop to promote angiogenesis and stimulate their accumulation.^126^ Interestingly, MDSCs could directly induce vascularization and tumor invasion by secreting matrix metalloproteinase-9 (MMP9) and differentiating into endothelial-like cells (ECs).^127^ MDSCs were also reported to support vascularization by means of exosomes which not only release proangiogenic factors but also prime target cells to acquire a proangiogenic phenotype.^128^ In addition, it was reported that granulocytic MDSCs-derived exosomal S100A9 promoted colorectal cancer (CRC) stemness in a HIF-1α-dependent manner.^129^ MDSCs from ovarian carcinoma patients were demonstrated to trigger miRNA101 expression and repress the corepressor gene C-terminal binding protein-2 (CtBP2) in cancer cells, subsequently leading to increased cancer cell stemness and metastatic potential.^130^ What’s more, in specimens from breast cancer patients, the level of MDSCs correlated with the presence of cancer stem-like cells (CSCs).^131^ Additionally, in colorectal carcinoma mouse model, elevated CXCL1 in the premetastatic liver was found to recruit CXCR2^+^ MDSCs to form a premetastatic niche, which in turn promoted liver metastases.^132^ Furthermore, PMN-MDSCs were reported to inhibit NK cells-mediated killing of circulating tumor cells (CTCs) and promote extravasation of CTCs through secreting IL-1β and MMPs in several mouse tumor models.^133^

### Differences in suppressive functions between PMN-MDSCs and M-MDSCs

PMN-MDSCs and M-MDSCs are distinct in terms of the phenotype and morphology, and in addition, they have unique although partially overlapping functional characteristics.^134^ Upregulated ARG1, iNOS, and ROS in MDSCs mainly mediate the immunosuppression on T cells. Among them, ARG1-induced suppression does not need cell-to-cell contact, while iNOS and ROS-mediated inhibition require proximity of T cells and MDSCs.^135^ Notably, the immunosuppressive activities of M-MDSCs primarily depend on ARG1, NO, and cytokines like IL-10 and TGFβ, which inhibit both antigen-specific and non-specific T-cell responses. However, PMN-MDSCs, whose functions mainly depend on high levels of ROS and RNS, primarily inhibit antigen-specific T-cell responses.^136^

Furthermore, the proportion and functional specialization of MDSCs differ in disparate tumor types and organs.^137,138^ In most mouse tumor models and cancer patients, PMN-MDSCs are predominantly detected in the peripheral lymphoid tissues and blood with relatively modest suppressive activities,^7,139,140^ while M-MDSCs are enriched in tumor tissues and rapidly differentiate into TAMs. In the TME, M-MDSCs are assessed to be more suppressive than PMN-MDSCs on a per-cell basis.^141,142^ These differences between PMN-MDSCs and M-MDSCs lead to more potent immunosuppressive properties of the total MDSCs population in tumor tissues compared with that in the periphery.

Recently, MDSCs subsets were reported to function in different spatiotemporal and sexual manners. In mouse tumor models, Ouzounova et al. demonstrated that tumor-infiltrated M-MDSCs promoted EMT/CSC phenotype to facilitate tumor cell dissemination from the primary sites. In contrast, pulmonary-infiltrating PMN-MDSCs supported the metastatic tumor growth through reverting EMT/CSC phenotype and promoting tumor cell proliferation.^143^ In glioblastoma (GBM) mouse models, proliferating M-MDSCs were predominant in the tumors of males, while a high PMN-MDSC/IL-1β gene signature was correlated to a poor prognosis in females. Moreover, chemotherapy was more efficient in targeting M-MDSCs in males, whereas IL-1 pathway inhibitor was more beneficial to inhibit PMN-MDSCs in females.^144^

Overall, the prevalence of a specific immunosuppressive mechanism depends on the MDSC subtypes, the tumor types/stages, and the organs/sites. Since the phenotype and function of MDSCs can change in response to local inflammatory factors, it is difficult to define particular markers predictive of their immune-suppressive properties.

## Therapeutic strategies targeting MDSCs in cancer

The fact that MDSCs play critical roles in tumor progression and metastasis has inspired the search for therapeutic strategies targeting these cells, which can be broadly classified into five categories: (1) inhibiting MDSCs expansion and recruitment; (2) promoting MDSCs differentiation into mature myeloid cells; (3) inhibiting MDSCs function; (4) inhibiting MDSCs metabolism; (5) depleting MDSCs directly. Herein, we will review this interesting field and also highlight some recent new studies.

### Inhibiting MDSCs expansion and recruitment

#### Anti-colony-stimulating factors

The development of MDSCs seems to be governed by the same growth factors modulating normal myelopoiesis.^145,146^ Recombinant human GM-CSF (rhGM-CSF) is used in the clinic to promote myeloid reconstitution following bone marrow transplantation or induction chemotherapy in AML patients. However, GM-CSF has also been found to stimulate the accumulation of MDSCs and impair the anticancer immune responses.^147^ In a clinical study, rhGM-CSF administration was proved to increase the MDSCs levels in the peripheral blood of patients with recurrent prostate cancer.^148^ Also, in a large randomized trial of advanced HNSCC patients treated with chemoradiotherapy, adjuvant G-CSF treatment was identified as a factor associated with poor prognosis due to decreased locoregional disease control.^149^ Moreover, G-CSF is a positive regulator of Bv8 (*Bombina variegata* 8) which not only mobilizes MDSCs from the bone marrow but also promotes angiogenesis in tumor.^150^ A lot of preclinical studies have reported that the blockade of GM-CSF/G-CSF signaling restricted the accumulation of MDSCs and restored antitumor immune response.^151,152^ In addition, myeloid cells in tumor sites always undergo the differentiation from monocytes/M-MDSCs towards TAMs, and this conversion is primarily mediated by CSF-1 and HIF-1α.^153^ CSF-1R blockade is an excellent approach to restrain the polarization towards M2 type macrophages in the TME.^154^ Previous studies have emphasized that improved effects were observed when CSF-1/CSF-1R blockade was combined with irradiation, paclitaxel, anti-VEGFR antibody, and immune checkpoint inhibitors in preclinical tumor models.^155,156^ In conclusion, CSFs blockade-based combination therapies require further verification as promising strategies to inhibit MDSCs expansion in cancer patients.

#### Anti-VEGF/VEGFR therapy

VEGF is an indispensable stimulator in MDSCs mobilization and expansion, and MDSCs in turn promote tumor angiogenesis and metastasis by secreting VEGF.^157^ VEGFR1 expressed on MDSCs is also accountable for their recruitment into tumor environment. In ovarian cancer patients, high VEGF expression in peritoneal dissemination was correlated with high MDSCs infiltration as well as an unfavorable prognosis.^158^ Thus, targeting the VEGF/VEGFR signaling pathway can reduce the recruitment of MDSCs and impede their angiogenesis-promoting effects.

The effects of widely applicated anti-VEGF/VEGFR therapies on MDSCs have been demonstrated in cancer patients. For instance, bevacizumab-based therapy significantly reduced the proportion of PMN-MDSCs in the peripheral blood of NSCLC patients.^159^ In a phase 0/I dose-escalation clinical trial (NCT02669173), low-dose, metronomic capecitabine combined with bevacizumab treatment significantly reduced circulating MDSCs levels in recurrent GBM patients and increased cytotoxic immune cells infiltration into the TME.^160^ Another study on patients with colorectal cancer reported that first line combination regimen of 5-FU, oxaliplatin, and bevacizumab (FOLFOX-bevacizumab) elicited a decrease of PMN-MDSCs in 15 of 25 patients, which was associated with a better survival outcome.^161^ However, in another study, bevacizumab treatment alone did not decrease the accumulation of MDSCs in the peripheral blood of renal cell carcinoma (RCC) patients.^96^ These discrepancies may be resulted from different choices in dosage, timing, or interval of drug administration. In fact, in preclinical studies, anti-VEGF/VEGFR agents could delete MDSCs in tumor, spleen, and in circulation in a dose-dependent manner.^162^

#### Anti-S100A8/A9

S100A8 (calgranulin A or myeloid-related protein 8, MRP8) and S100A9 (calgranulin B or myeloid-related protein 14, MRP14) are small molecular calcium-binding proteins that play crucial roles in cancer development, and thus are considered as diagnostic markers and novel targets for cancer therapy.^163,164^ MDSCs express both S100A8/A9 and the corresponding receptors RAGE, working as a positive feedback loop to recruit MDSCs and enhance their immunosuppressive function.^165^ Inhibiting S100A8/A9 has been indicated to restrain tumor growth by reducing the accumulation of MDSCs in several mouse tumor models.^166–168^ Tasquinimod is an oral agent that can bind to S100A9 and in turn block the interaction between S100A9 and its sensors, including RAGE and TLR4.^169^ Many studies have established that tasquinimod treatment in cancer can lead to depletion of blood monocytes, reduction of MDSCs infiltration into tumor sites, and induction of TAMs to M1 polarization.^170^ In a phase II trial, tasquinimod treatment improved PFS in patients with metastatic castration-resistant prostate cancer (mCRPC) by reducing the recruitment of MDSCs and inhibiting metastasis.^171,172^ Recently, in a phase III clinical trial, tasquinimod treatment in mCRPC patients led to considerably longer radiologic PFS compared with the placebo group, but no influence on the OS was observed.^173^ Contradictorily, another phase II clinical trial (NCT01743469) aiming to examine the effectiveness of tasquinimod in patients with advanced hepatocellular, gastric, ovarian, and renal cell carcinomas revealed no efficiency of tasquinimod on any of these tumor types.^174^ Together, these findings propose S100A8/A9 as advisable targets to ameliorate MDSCs-mediated immunosuppression in cancer. However, further exploration on the efficacy of S100A8/A9-targeting strategies is wanted.

#### Anti-IL-1β

IL-1β contributes to tumor initiation and progression mainly by inducing chronic non-resolved inflammation, promoting angiogenesis, as well as driving MDSCs expansion and migration.^175^ In the peripheral blood of advanced melanoma patients, an elevated frequency of IL-1β was positively correlated with the level of M-MDSCs.^176^ And it was reported that M-MDSCs in the premetastatic lungs of tumor-bearing mice could produce IL-1β to increase E-selectin expression, which in turn promoted the arrest of tumor cells on endothelial cells.^177^ On the other hand, the NLRP3 (NOD-like receptor family pyrin domain containing 3) inflammasome is one of the most well recognized inflammasomes that promotes IL-1β maturation and secretion.^178^ In a study from both HNSCC patients and mouse models, the level of IL-1β was increased in the peripheral blood, while the NLRP3 inflammasome was overexpressed in the tumor tissues. Currently, several agents are available to inhibit IL-1, which include IL-1Ra (anakinra), IL-1β specific antibodies (canakinumab), as well as inflammasome inhibitors.^179–181^ Notably, multiple cancer therapeutic agents such as chemotherapeutic drugs, MAPK inhibitors, and BRAF V600E inhibitor (BRAFi) have been reported to either increase the expression of IL-1β or activate inflammasomes in myeloid cells,^182,183^ causing unwanted side effects. In this regard, IL-1β blockade may generate adjunctive effects when combined with chemotherapies or other treatments in cancer.^184^

#### Anti-CCL2/CCR2

The CCL2/CCR2 pathway contributes significantly to the migration of M-MDSCs to tumor sites. Moreover, overexpressed CCL2 has been found in many cancers, which is always associated with disease progression.^50,185,186^ In preclinical mouse tumor models, the combinations of CCL2/CCR2 blockade with radiotherapy, immunotherapy, and targeted therapy have shown synergistic and improved antitumoral effects, along with decreased tumor-associated MDSCs as well as increased tumor-infiltrating lymphocytes.^187–189^ In a phase I dose-escalation study, patients with primary breast cancer were administered safely with CCL2 inhibitor propagermanium (PG), which was expected to exert anti-metastatic potential.^190^ However, a humanized monoclonal antibody (mAb) against CCL2 CNTO888 showed no antitumor activity as a single agent in mCRPC patients.^191^ The limited therapeutic efficacy of CNTO888 may be ascribed to transient neutralization of free-CCL2, followed by significant accumulation of total CCL2 in the circulation.^191^ Additionally, CCR2-targeting strategies also show efficacy in cancer management. Results from a phase Ib trial revealed that, PF-04136309 (a small molecule inhibitor of CCR2) in combination with the chemotherapy regimen FOLFIRINOX treatments in patients with pancreatic ductal adenocarcinoma inhibited the migration of inflammatory monocytes from the bone marrow, leading to decreased TAMs and higher tumor control rates.^192^ Moreover, pancreatic cancer patients treated with CCR2 inhibitor CCX872 coupled with FOLFIRINOX had longer overall survival compared with those in FOLFIRINOX monotherapy group.^193^ Currently, another CCR2 inhibitor BMS-813160 is being tested in clinical trials combined with immunotherapy or chemotherapy for the treatment of solid tumors (Table 1).

**Table 1:** Summary of clinical trials targeting MDSCs in cancer.

	Target	Drug name	Indications	Phase	Last reported status	NCT number
Inhibiting expansion and recruitment	G-CSF	G-CSF, Cabazitaxel	Prostate cancer	III	Recruiting	NCT02961257
	VEGF	Bevacizumab, Capecitabine	Glioblastoma	I	Recruiting	NCT02669173
	VEGF/ VEGFR	Bevacizumab, Pazopanib	Renal cell carcinoma/cancer	I/II	Recruiting	NCT01684397
	VEGF	Bevacizumab, Anakinra, LV5FU2	Colorectal cancer	II	Completed	NCT02090101
	VEGFR	Pazopanib	Prostate adenocarcinoma	II	Completed	NCT01832259
	VEGFR	Cabozantinib	Prostate cancer	II	Recruiting	NCT03964337
	EGFR	Cetuximab, Edodekin alfa	Head and neck carcinoma	I/II	Active, not recruiting	NCT01468896
	EGFR	Cetuximab, Cyclophosphamide	Head and neck cancer	II	Completed	NCT01581970
	S100A9	Tasquinimod	Advanced cancer	II	Completed	NCT01743469
	CXCR1/2	Reparixin, Paclitaxel	Metastatic breast cancer	II	Completed	NCT02370238
	CXCR1/2	Reparixin, Paclitaxel	Metastatic breast cancer	I	Completed	NCT02001974
	CCR2	CCX872-B	Pancreatic cancer	I	Active, not recruiting	NCT02345408
	CCR2	MLN1202	Cancer	II	Completed	NCT01015560
	CCR2	PF-04136309, Chemotherapy	Pancreatic adenocarcinoma	I	Completed	NCT01413022
	CXCR2	AZD5069, Enzalutamide	Prostate cancer	I/II	Recruiting	NCT03177187
	CCR5	Leronlimab + Carboplatin	Triple negative breast neoplasms	I/II	Recruiting	NCT03838367
	IL-8	HuMax-IL8	Solid tumor	I	Completed	NCT02536469
	PI3K	Duvelisib, Ibrutinib	Lymphocytic leukemia	II	Recruiting	NCT04209621
	PI3K	Idelalisib	Hodgkin lymphoma	II	Completed	NCT01393106
Promoting differentiation	STAT3	AZD9150	Hepatocellular carcinoma	I	Completed	NCT01839604
	STAT3	IONIS-STAT3Rx	DLBCL lymphoma	I/II	Completed	NCT01563302
	TLR7	Imiquimod, Abraxane	Breast cancer	II	Completed	NCT00821964
		Curcumin	Breast cancer	I	Recruiting	NCT03980509
		Curcumin	Prostate cancer	III	Recruiting	NCT03769766
		Curcumin	Breast cancer	II	Completed	NCT03072992
		β-glucan	Oral cavity carcinoma	Not applicable	Active, not recruiting	NCT04387682
		β-glucan	NSCLC	Not applicable	Recruiting	NCT00682032
Inhibiting function	PDE5	Tadalafil	Head and neck cancer	Not applicable	Completed	NCT00843635
	PDE5	Tadalafil	Head and neck carcinoma	II	Completed	NCT00894413
	PDE5	Tadalafil	Head and neck carcinoma	II	Completed	NCT01697800
	PDE5	Sildenafil, Chemotherapy	NSCLC	II/III	Completed	NCT00752115
	NRF2	Omaveloxolone	NSCLC, Melanoma	I	Completed	NCT02029729
	H2R	Ranitidine	Cancer	IV	Active, not recruiting	NCT03145012
Inhibiting metabolism	IDO	Indoximod, Docetaxel, Paclitaxel	Breast cancer	II	Completed	NCT01792050
	CD73/A2AR	MEDI9447, AZD4635	Carcinoma, NSCLC	I/II	Active, not recruiting	NCT03381274
Depleting MDSCs	CD33	GTB-3550 TriKE™	Leukemia	I/II	Recruiting	NCT03214666
		Gemcitabine	Pancreatic cancer	II	Completed	NCT01019382
		Cyclophosphamide, Docetaxel, Doxorubicin, Oxidized glutathione	Breast cancer	II	Completed	NCT00499122
	TRAIL-R2	DS-8273a	Solid tumor, Lymphoma	I	Completed	NCT02076451
Other therapies		Octreotide acetate	Neuroendocrine tumor	II	Active, not recruiting	NCT04129255
		Qingshu-Yiqi-Tang	Carcinoma, NSCLC	II/III	Recruiting	NCT01802021
		Soy bread diet	Prostate adenocarcinoma	II	Recruiting	NCT03654638

One of the plausible reasons for the dissatisfactory results from current clinical trials is that neither CCL2 neutralizing antibodies nor CCR2 inhibitors can effectively block the CCL2-CCR2 axis for a long time.^191,194^ In addition, the infiltration of MDSCs into tumor sites is controlled by various alternative factors such as the ligands of CCR5, hence therapeutic blockade with a single particular chemokine inhibitor has limited effects.^53^ Although targeting chemokine receptors is more efficient because one receptor may interact with several chemokines, we should pay attention that many of the CC chemokines can simultaneously induce the recruitment of APCs and TILs into tumor tissues.^195^ Nevertheless, targeting the CC chemokine/receptor axis exhibits great potential for cancer therapy, particularly in combination with immunotherapies.

#### Anti-CXCLs/CXCR1/2

In tumor-bearing hosts, the activated CXCLs/CXCR1/2 axis plays an important role in supporting immune evasion and tumor progression partially through promoting neutrophils and PMN-MDSCs recruitment.^196^ Further, traditional anticancer treatments such as chemotherapy and radiotherapy have been found to induce inflammatory CXCLs release, which in turn lead to therapy resistance. The combination of chemotherapies with the CXCLs/CXCR1/2 axis blockade showed synergistic effects in enhancing antitumor activity in preclinical tumor models.^197,198^ Anti-CXCLs/CXCR1/2 therapies have also been reported to improve the efficacy of immune checkpoint inhibitors (ICIs), adoptive transferred engineered T cells and NK cells in various tumor models through abrogation of PMN-MDSCs trafficking into tumor sites.^51,199,200^ To date, several CXCR1/2 inhibitors have been assessed in clinical trials for cancer treatment, such as Reparixin, Navarixin, AZD5069, and SX-682.^201^ In addition, ABX-IL8 and HuMax-IL8 are two well-investigated humanized mAbs targeting IL-8 (CXCL8 is also known as IL-8 in human).^202^ Importantly, HuMax-IL8 has been confirmed to be safe and tolerable in patients with locally advanced solid tumors, and now is under evaluation in a phase Ia/II study in combination with nivolumab (NCT03400332).^203^ In all, blockade of the CXCLs/CXCR1/2 axis exhibits limited direct antitumor effects, and therefore, combining the CXCLs/CXCR1/2 axis inhibition therapy with chemotherapy, anti-angiogenesis therapy, and immunotherapy in cancer treatment is practicable. Additionally, the level of serum IL-8 can be used as a valuable diagnostic biomarker to select patients in whom these combinations should be evaluated.^204^

### Promoting MDSCs differentiation into mature myeloid cells

#### STAT3 inhibitors

Constitutive phosphorylation of STAT3 is a pivotal molecular event that regulates the expansion and immunosuppressive function of MDSCs in cancer,^205^ besides, STAT3 also prevents the differentiation of IMCs into mature DCs and macrophages.^206^ Thus, STAT3 can serve as an attractive therapeutic target to reduce MDSCs for cancer management.^207^ Oral treatment with cucurbitacin B (a selective inhibitor of JAK2/STAT3) daily for seven consecutive days was found to decrease IMCs and simultaneously increase the levels of mature myeloid cells in the peripheral blood of patients with advanced lung cancer.^208^ Furthermore, a phase Ib trial (NCT01563302) revealed that systemic administration of AZD9150, an antisense oligonucleotide inhibitor of STAT3, reduced the levels of peripheral PMN-MDSCs in patients with diffuse large B-cell lymphoma (DLBCL).^209^ More interestingly, accumulating studies have indicated the rationale and feasibility of STAT3 inhibition in combination with immunotherapy in cancer treatment. In mouse liver metastasis tumor models, STAT3 inhibitors markedly promoted Bax-dependent apoptosis of MDSCs and further enhanced the antitumor efficiency of chimeric antigen receptor T-cell (CAR-T) therapy.^210^ Currently, a phase II clinical trial testing AZD9150 coupled with anti-PD-L1 mAb (MEDI4736) and anti-CTLA-4 mAb (tremelimumab) in patients with advanced solid tumors and relapsed metastatic HNSCC is ongoing (NCT02499328). Therefore, targeting STAT3 signaling is along the encouraging direction of tumor immunotherapy.

#### All-trans retinoic acid (ATRA)

ATRA is a derivative of vitamin A with agonistic activity towards retinoid-activated transcriptional regulators (RARα and RARβ). These regulators consecutively activate downstream signals and subsequently induce the maturation of primitive myeloid cells into fully differentiated (less-immunosuppressive) variants.^211^ Acute promyelocytic leukemia is considered as one of the most well-defined targeted cancer types of ATRA. Recent studies on other cancer types have highlighted that ATRA can stimulate the differentiation of MDSCs into mature DCs, macrophages, and granulocytes.^211,212^ The ATRA-induced differentiation of MDSCs deals with increased glutathione synthase (GSS) and glutathione (GSH) production in MDSCs, which neutralize ROS and drive the myeloid-cell differentiation.^213^ In patients with mRCC, administration of ATRA with high plasma concentration (>150 ng/mL) abrogated MDSCs-mediated immunosuppression by promoting their differentiation into APC precursors, effectively improving T cells-induced cytotoxicity on tumor cells.^29^ ATRA has also been utilized to enhance the effects of conventional chemotherapeutic agents and immunotherapies in cancer.^214^ Data from a clinical trial in patients with extensive-stage small-cell lung cancer (SCLC) elucidated that the combination of ATRA with vaccination (DCs transduced with wild-type p53) depleted MDSCs from peripheral blood substantially and enhanced the immune response to vaccination.^215^ Another trial (NCT02403778) found that the addition of ATRA to the standard ipilimumab therapy in patients with stage IV metastatic melanoma considerably reduced the number of circulating MDSCs compared with ipilimumab therapy alone.^216^ Although ATRA as a single agent is less effective in solid tumors, it might augment immune response and prolong the survival of patients by inducing the differentiation of MDSCs.

#### Toll-like receptors (TLRs) agonists

TLRs are type I transmembrane proteins that can recognize both endogenous and exogenous damage-associated and pathogen-associated molecular patterns (DAMPs and PAMPs), inducing innate immune responses. Many clinical trials have indicated that synthetic oligodeoxynucleotides (ODN) containing unmethylated cytosine-phosphorothioate-guanine (CpG) motifs, agonists for TLR9, have antitumoral immune activity as therapeutic vaccine adjuvants.^217^ Recent papers have documented that the anticancer efficiency of CpG ODN acted partially by inducing the differentiation and maturation of MDSCs.^218,219^ IFN-α produced by plasmacytoid DCs upon CpG stimulation has been identified as a key effector to promote the maturation of PMN-MDSCs.^220^ Intriguingly, the CpG-STAT3siRNA conjugate (ODN coupled to STAT3 siRNA) strategy could trigger TLR9 immunostimulation and eliminate the negative effects of STAT3 concomitantly in myeloid cells.^221^ Studies have concluded that PMN-MDSCs expressing high levels of TLR9 and STAT3 accumulated in the circulation and tumor site of prostate cancer patients, and CpG-STAT3siRNA abrogated the immunosuppressive effects of these MDSCs effectively.^222^

TLR7/8 agonists also serve as monotherapy or synergize with immunotherapeutic approaches to enhance antitumor effects by inducing MDSCs to acquire non-suppressive capability.^223,224^ In a phase Ib trial (NCT02124850), fourteen patients with primarily diagnosed HNSCC were enrolled and treated with TLR8 agonist motolimod plus cetuximab preoperatively. The findings revealed that fewer MDSCs and increased M1 monocytes were found in tumor tissues.^225^ Folate-linked TLR7 agonists could also induce the abrogation of MDSCs/TAMs-mediated immunosuppression and enhance T cell infiltration, improving survivals of mouse tumor models.^226^

Polyinosinic-polycytidylic acid (poly I:C, a synthetic double-stranded RNA ligand for TLR3) is utilized as an adjuvant to enhance antitumor immunity.^227^ Poly I:C also exhibits the potential to decrease the frequency of MDSCs and abrogate their immunosuppressive function.^228^ In a B16 tumor model, after poly I:C administration, MDSCs produced increased IFN-α through the activation of the mitochondrial antiviral signaling protein (MAVS) pathway and sequentially motivated NK cells, leading to delayed tumor growth.^229^ Currently, poly I:C is mainly used in combination with other anticancer therapies in preclinical studies, including irradiation,^230^ cancer vaccine, and CAR-T therapy.^231^ Nevertheless, the effect of poly I:C in cancer patients requires further investigation.

#### Other potential therapies

Like ATRA, vitamin D3 can induce the differentiation of MDSCs and improve the antitumor immune responses.^28,232^ Treatment with 1α,25(OH)_2_D_3_ in HNSCC patients before surgery reduced the frequency of immune inhibitory CD34^+^ progenitor cells while increased the maturation of DCs in tumor tissues.^233^ In another study, HNSCC patients administrated with 1α,25(OH)_2_D_3_ had increased intra-tumoral CD4^+^ and CD8^+^ T cells and a lengthier tumor progression free time compared to untreated patients.^234^

Curcumin,^235^ icariin (ICA),^236^ and β-glucans^237^ have also been reported to promote the differentiation of MDSCs as well as reduce the associated immunosuppression in preclinical tumor models. For instance, curcumin treatment polarized MDSCs to an M1-like phenotype with increased CCR7 expression and decreased dectin 1 expression in vivo and in vitro.^238,239^ Additionally, treatment of NSCLC patients with particulate β-glucan for two weeks reduced the levels of PMN-MDSCs in the peripheral blood.^240^ Further research found that whole β-glucan particles (WGPs) could inhibit nuclear factor I-A (NFIA) expression in PMN-MDSCs.^241^ Based on this concept, intensive studies are wanted to identify the therapeutic potential of above-mentioned compounds, especially in cancer patients.^242,243^

### Inhibiting MDSCs function

#### COX-2/PGE2/EP axis inhibitors

The abnormally activated COX-2/PGE2/EP pathway has recently emerged as an attractive therapy target in tumor-bearing hosts. This pathway was demonstrated to enhance MDSCs accumulation,^244^ maintain their suppressive function,^245,246^ and regulate the PD-L1 expression on tumor-infiltrating MDSCs.^247^ Particularly, PGE2 has been proved to improve the production of CXCL12, causing the CXCL12-CXCR4-mediated attraction of MDSCs into the TME.^244^ In addition, tumor-derived PGE2 was reported to mediate the activation of nuclear p50/NF-κB in M-MDSCs, diverting their response to IFN-γ towards NO-mediated immunosuppression and reducing their TNFα production.^248^ On the other hand, there is a positive feedback loop between PGE2 and COX-2 in MDSCs. PGE2 derived from tumor or stroma cells induces high levels of COX-2 expression in MDSCs through prostaglandin E (EP) 2/EP4 receptors, and COX-2 consecutively initiates the autocrine production of endogenous PGE2 and stabilizes the suppressive functions of MDSCs.^249^ Recently, a novel signaling circuit has been demonstrated in colorectal cancer. The downregulation of RIPK3 (receptor-interacting protein kinase 3) in tumor-infiltrating MDSCs potentiated COX-2-mediated PGE2 production which further reduced RIPK3 and promoted the immunosuppressive activity of MDSCs.^250^

Multiple preclinical studies have explored the effects of the COX-2/PGE2/EP axis blockade on the development of MDSCs in cancer.^251,252^ For example, dietary treatment of celecoxib decreased local and systemic accumulation of all MDSC subtypes and reduced the levels of ROS and NO in tumor-bearing mice.^253^ Moreover, combination treatment of anti-CD40 agonist and celecoxib decreased the ARG1 expression in MDSCs and increased the survival of GL261 glioma-bearing mice, compared with monotherapy alone.^254^ Current therapies targeting COX-2 using nonsteroidal anti-inflammatory drugs (NSAIDs) or COX-2 inhibitors have severe adverse effects because of global prostanoid suppression, therefore, targeting the downstream molecules of the PGE2 pathway can also be a potential approach.^255^ Results from a phase I clinical trial (NCT02540291) in patients with advanced solid tumors showed that oral administration of E7046, an EP4 inhibitor, significantly enhanced tumor infiltration of CD3^+^ and CD8^+^ T cells, but the levels of MDSCs in these patients were not reported. Accumulating evidence has shown that EP4 antagonism should be investigated further as a promising strategy for cancer treatment, particularly in combination with chemotherapy, endocrine therapy, or immune-based therapy.^256,257^

#### Phosphodiesterase 5 (PDE5) inhibitors

PDE5 inhibitors (such as sildenafil, tadalafil, and vardenafil) have been routinely applied for the treatment of erectile dysfunction, benign prostatic hyperplasia, cardiac hypertrophy, and pulmonary hypertension.^258^ These inhibitors were also reported to downregulate the expression of ARG1, iNOS, and IL-4Ra in MDSCs via increasing the intracellular cyclic guanosine monophosphate (cGMP) concentrations, thus making MDSCs less immunosuppressive.^259,260^ One possible molecular mechanism for these effects is that cGMP destabilizes iNOS mRNA by reducing the ubiquitous mRNA binding protein. Another possibility is that high levels of cGMP reduce the concentration of cytosolic Ca^2+^ and thus inhibit the activity of calcium-dependent protein kinase C, which consecutively prevents the upregulation of IL-4Rα and ARG1 in MDSCs.^261^

In an open-label, dose de-escalation trial, tadalafil treatment in metastatic melanoma patients was proved to be safe and well-tolerated, with clinically stable patients displaying significant infiltration of CD8^+^ T cells and reduction of MDSCs in metastasis lesions.^262^ Another study (NCT00843635) showed that, in HNSCC patients, tadalafil therapy considerably reduced the concentrations of both MDSCs and Tregs in the blood and tumor.^263^ The activity of tadalafil was maximized at an intermediate dose (10 mg/d) compared with a high dose (20 mg/d), indicating that high dosages might negatively affect antitumor immunity by increasing the production of intracellular cAMP. Similar findings were also reported in another clinical trial (NCT00894413), in which tadalafil treatment in HNSCC patients augmented systemic and tumor-specific immunity, reduced peripheral MDSCs numbers, and decreased ARG1 and iNOS in total MDSCs.^264^ However, even though PDE5 inhibitors can induce enhanced CTL responses, such treatment alone is unlikely to eliminate tumors completely, and on this basis, a combination with other therapies is a rational choice. For instance, a recent study showed that tadalafil combined with lenalidomide, dexamethasone, and clindamycin generated a durable clinical response in a patient with end-stage multiple myeloma, along with decreased expression levels of IL-4Ra, ARG1, and iNOS in bone marrow M-MDSCs.^259^ Moreover, a phase I trial (NCT01342224) testing tadalafil and a telomerase vaccine (GV1001) alongside gemcitabine in patients with locally advanced pancreatic adenocarcinoma is ongoing.

#### Epigenetic regulators

Histone deacetylase inhibitors (HDACis) are important epigenetic regulators.^265^ Recent studies in preclinical mouse tumor models have shown that HDACis can significantly reduce ARG1, iNOS, and COX-2 expression in MDSCs, thus promoting the efficiency of immunotherapeutic agents.^266,267^ A study on EL4 lymphoma and LLC (Lewis lung carcinoma) mouse models reported that the selective class I HDACi entinostat reduced the immunosuppressive activity of PMN-MDSCs. Whereas, M-MDSCs expressed high levels of class II HDAC6, and inhibition of HDAC6 using ricolinostat decreased the immunosuppressive activity of M-MDSCs.^268^ Furthermore, adjuvant epigenetic therapies using entinostat and low-dose 5-azacytidine (DNA methyltransferase) disrupted the formation of premetastatic niche after surgery in pulmonary metastases mouse models. The underlying mechanism was that epigenetic therapies not only inhibited MDSCs trafficking by downregulating CCR2 and CXCR2 but also induced MDSCs differentiation towards a more-interstitial macrophage-like phenotype.^269^ A phase II clinical trial confirmed that the combination of entinostat and pembrolizumab provided a clinical meaningful benefit for patients with immune checkpoint inhibitor-resistant NSCLC.^270^ Another clinical trial testing the combination treatment of nivolumab, 5-azacytidine, and entinostat in NSCLC patients (NCT01928576) is ongoing. Nevertheless, HDACis were reported to have both stimulatory and detrimental effects on immune cells, depending on immune cell types, cell activation status, and the class of HDACis.^265^ Therefore, further studies are required to explore the mechanisms of rational combination of immunotherapy with HDACis to develop effective therapies for cancer patients.

#### Nuclear factor erythroid 2-related factor 2 (Nrf2) pathway activator

Nrf2 is a ubiquitous master transcription factor which modulates several genes to attenuate oxidative stress. Nrf2 also contributes to the clearance of ROS in MDSCs and enables MDSCs to survive in the noxious TME.^271^ Systemic Nrf2-deletion or myeloid lineage Nrf2-deficiency in tumor-bearing mice could cause aberrant ROS accumulation in MDSCs, leading to increased susceptibility to cancer metastasis.^272,273^ The synthetic triterpenoid CDDO-Me (bardoxolone methyl, RTA402) is used for the treatment of chronic kidney disease, cancer, and other diseases.^274^ Recent studies have found that CDDO-Me could inhibit the immunosuppressive capacity of MDSCs by activating Nrf2 and inhibiting ROS generation in MDSCs. Nagaraj et al. reported that CDDO-Me treatment in tumor-bearing mice inhibited the suppressive activity of splenic MDSCs, resulting in decreased tumor growth.^275^ In addition, they performed a phase I clinical trial (NCT00529113) in which pancreatic adenocarcinoma patients were intravenously administered with gemcitabine on days 1, 8, and 15 weekly and CDDO-Me orally once daily for 21 days. Analysis showed that CDDO-Me had no effect on MDSCs levels in the peripheral blood, but it significantly improved the immune response in these patients.^275^ However, it is not clear whether the level of Nrf2 in MDSCs from peripheral lymphoid organs or tumor tissues is different.^276^ In addition to its anti-oxidative activity, Nrf2 may also contribute to a context-dependent regulation of MDSCs.^277^ In general, Nrf2 is a potential target in cancer treatment which deserves further investigation.

#### Other potential therapies

Nitroaspirin or NO-releasing aspirin, a compound covalently linking a NO-releasing moiety and a classic aspirin molecule,^278^ was reported to inhibit ARG1 and iNOS production in MDSCs. When co-administered with a DNA vaccine, nitroaspirin (NCX 4016) inhibited the function of MDSCs and improved the survival of CT26 colon carcinoma mouse model.^279^ Mechanistically, analysis showed that the NO release contributed to iNOS inhibition, whereas the aspirin spacer portion caused the ARG-dependent inhibitory effect.^279^

NOV-002 (oxidized glutathione), a glutathione disulfide mimetic with the ability to induce S-glutathionylation, has been examined effective in patients with platinum-refractory ovarian cancer and advanced NSCLC.^280,281^ A preclinical study in mouse tumor model demonstrated that NOV-002 ameliorated cytotoxic chemotherapy-induced hematopoietic and immune suppression partially through inhibiting ROS production in MDSCs.^282^ In a phase II clinical trial (NCT00499122), breast cancer patients were treated with a combination of NOV-002 and preoperative chemotherapy (doxorubicin, cyclophosphamide, and docetaxel). Analysis showed that patients with higher pathologic complete response (pCR) rates had lower levels of MDSCs in blood.^283^

### Inhibiting MDSCs metabolism

#### Targeting fatty acid metabolism

**Fig. 5:**
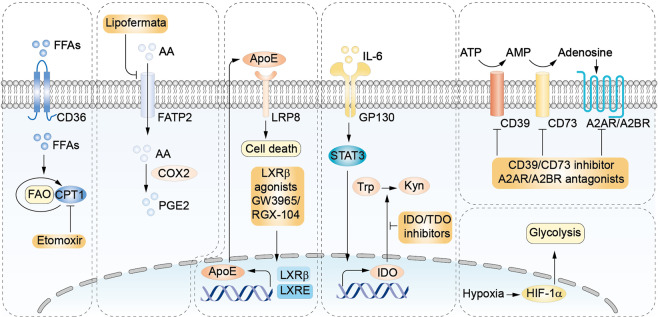
Targeting MDSCs by interrupting their metabolism. MDSCs are characterized by high free fatty acids (FFAs) uptake and fatty acid oxidation (FAO). Carnitine palmitoyltransferase 1 (CPT1), the first rate-limiting enzyme in the FAO cycle, can be inhibited by etomoxir. FATP2 mediates the uptake of arachidonic acid (AA) which subsequently promotes the synthesis of PGE2 in MDSCs. Lipofermata selectively inhibits FATP2. LXR agonists promote the transcriptional upregulation of apolipoprotein E (ApoE) which subsequently reduces MDSCs survival by binding to low-density lipoprotein receptor-related protein 8 (LRP8) on MDSCs. IDO and tryptophan 2,3-dioxygenase (TDO) catalyze the first rate-limiting step in the conversion of L-tryptophan to L-kynurenine. IL-6 upregulates the transcription of IDO promoter in MDSCs through STAT-3 activation. IDO/TDO inhibitors attenuate the suppressive capability of MDSCs. Adenosine-generating axis and its receptor A2AR/A2BR are also metabolic targets to inhibit MDSCs. MDSCs exhibit upregulated glycolysis which is regulated by HIF-1α. CD36, fatty acid translocase; FATP2, fatty acid transport protein 2; LXR, Liver-X receptors; Trp, tryptophan; Kyn, kynurenine.

MDSCs are characterized by high uptake of free fatty acids (FFAs) and increased expression of key fatty acid oxidation (FAO) enzymes (Fig. 5).^284,285^ Selectively targeting fatty acid metabolism of MDSCs can impede the associated immune suppression. Etomoxir, a specific inhibitor of carnitine palmitoyltransferase 1 (CPT1, the first rate-limiting enzyme in FAO pathway), significantly delayed tumor growth in several mouse tumor models in a T-cell-dependent manner. Furthermore, the combination of etomoxir with low-dose chemotherapy completely abrogated the immunosuppressive function of tumor-infiltrating MDSCs.^141^ Besides, a previous study reported that GM-CSF signaling induced the overexpression of fatty acid transport protein 2 (FATP2) in PMN-MDSCs through activation of STAT5, and FATP2 in turn modulated the immunosuppressive function of PMN-MDSCs through uptake of arachidonic acid and synthesis of PGE2. The selective FATP2 inhibitor lipofermata, alone or in combination with checkpoint inhibitors, inhibited the activity of PMN-MDSCs and substantially delayed tumor progression in mice models.^286^

Transcription factors liver-X receptors (LXRβ and LXRα) are additional lipid metabolism-related targets in MDSCs. LXRs belong to the nuclear hormone receptor family and are involved in the transcriptional activation of genes associated with cholesterol, fatty acid, and glucose metabolism. LXR agonists have the potential to inhibit tumor proliferation and survival, as well as to elicit significant antitumor immunity effects.^287^ A recent study reported that the LXRβ agonists, GW3965 and RGX-104, reduced the MDSCs levels in several mouse tumor models.^288^ Mechanistically, LXR agonism upregulated the transcriptional target apolipoprotein E (ApoE), which bound to low-density lipoprotein receptor-related protein 8 (LRP8) on MDSCs to reduce MDSCs survival. Moreover, the combination of RGX-104 and various immune-based therapies (such as CAR-T and anti-PD-1 therapies) elicited robust antitumor immunity responses in mouse tumor models.^289^ A multicenter dose-escalation phase I trial of RGX-104, alone or in combination with chemotherapy/immune checkpoint inhibitor in patients with lymphomas or metastatic solid tumors, is ongoing (NCT02922764). Primary data have shown that the combined therapies induced MDSCs depletion and CTLs activation in cancer patients.

#### Targeting glycolysis

MDSCs upregulate glycolysis enzymes and exhibit high glycolytic rate, which protect them from apoptosis and result in their accumulation in cancer patients.^290^ Moreover, it was shown that tumor-infiltrating M-MDSCs had upregulated mTOR phosphorylation and higher glycolysis than splenic M-MDSCs in mouse tumor models.^291^ And mTOR inhibitor rapamycin decreased the glycolysis, the immunosuppressive activities, and the percentage of tumor-infiltrating M-MDSCs in tumor-bearing mice.^292^ In addition, the glycolytic pathway of MDSCs is modulated by HIF-1α which can be inhibited by AMP-activated protein kinase (AMPK) activation. And AMPK activation also can inhibit immune-related NF-κB, JAK-STAT, CHOP, and C/EBP pathways which are involved in the expansion and activation of MDSCs.^293^ Studies reported that pharmacological activation of AMPK by metformin inhibited the aggregation and immunosuppressive ability of MDSCs in tumor-bearing mice.^294,295^ Moreover, metformin therapy was reported to abrogate the inhibitory activity of MDSCs in ovarian cancer patients through downregulating the expression and the extracellular enzyme activities of CD39 and CD73 in MDSCs.^296^ However, another study in tumor-bearing mice reported that conditional deletion of Prkaa1 in myeloid cells or systemic inhibition of AMPKα both reduced the immunosuppression of MDSCs and delayed tumor growth.^297^ Therefore, more studies should be conducted to investigate the role of glycolysis in modulating the immunosuppressive effects of MDSCs, especially in the context of tumors.

#### Targeting tryptophan catabolism

The tryptophan-kynurenine-aryl hydrocarbon receptor (Trp-Kyn-AhR) pathway is a generally accepted mediator of immunosuppression in tumors.^298^ IDO and tryptophan 2,3-dioxygenase (TDO) catalyze the first rate-limiting step in the conversion of L-tryptophan to N-formyl-L-kynurenine. IDO is highly expressed in many human cancers, which is positively associated with tumor stage and tumor metastatic status.^299^ Besides, IDO is highly expressed in tumor-infiltrating fibroblasts, endothelial cells, and immune cells such as MDSCs.^300^ Activated IDO has multifaceted effects, such as inhibition of T and NK cells, recruitment and activation of Tregs and MDSCs, and induction of angiogenesis and tumor metastasis.^301^ Interestingly, a study found that the IDO1 expression in tumor cells of triple-negative breast cancer (TNBC) patients was directly correlated with the level of circulating e-MDSCs.^302^ Moreover, it was reported that IL-6 triggered the transcriptional upregulation of IDO promoter in breast cancer-derived MDSCs through STAT3 signaling, and in breast cancer patients treated with neoadjuvant chemotherapy, the frequency of IDO^+^ MDSCs was positively correlated with the level of Tregs in tumors but was negatively associated with the outcome of patients.^303^

IDO inhibitors including epacadostat, navoximod, EOS200271, and BMS-986205 have been tested to be safe and well tolerated in patients with advanced solid malignancies.^304^ Clinical trials testing IDO inhibitors combined with immune checkpoint inhibitors in cancer patients are ongoing, with early results indicating that the combinatory therapies are effective and well tolerated.^305,306^ However, the combination therapy of epacadostat and pembrolizumab in a phase III trial of patients with unresectable or metastatic melanoma (NCT02752074) failed to meet its primary end point.^307^ Further, IDO inhibitors in combination with radiotherapy, chemotherapy, and antitumor vaccines are also being tested in clinical trials.^308^ Currently, dual IDO–TDO inhibitors and novel Trp-Kyn-AhR pathway inhibitors such as Kyn-degrading enzymes, direct AhR antagonists, and tryptophan mimetics are being explored.^309^

#### Targeting adenosine metabolism

Metabolic pathway of immunosuppressive adenosine is a key mediator to regulate tumor immunity.^108^ Inhibition of extracellular adenosine (eADO)-generating enzymes and/or eADO receptors can improve antitumor immunity through various mechanisms, such as promotion of T cell and NK cell function, suppression of MDSCs, and stimulation of antigen presentation. Several agents targeting distinct components of the CD39-CD73-A2A/BR pathway are currently being tested in early phase clinical trials as monotherapy or in combination with immunotherapies, with preliminary data indicating good tolerability.^310^ Additionally, blockade of this pathway can be combined with therapies which promote hypoxia within the TME such as radiation therapy and chemotherapy.^311,312^ Furthermore, other potential strategies including co-inhibition of CD39 and CD73,^313,314^ dual inhibitor of A2AR and A2BR, and co-inhibition of A2AR and CD73 are currently being explored.^315^

### Depleting MDSCs

#### Low-dose chemotherapy

Chemotherapeutic agents have direct cytotoxicity on tumor cells, and also exert immunomodulatory effects to selectively eliminate MDSCs and reduce their immunosuppression.^316^ Gemcitabine and fluorouracil (5-FU) are two generally recognized cytotoxic agents which deplete MDSCs in cancer-bearing individuals.^317^ Multiple studies have demonstrated that the combination of chemotherapeutic drugs with immunotherapies could decrease MDSC numbers and lead to synergistic benefits on the survival of cancer patients.^160,316^ Besides, gemcitabine pretreatment could enhance the efficacy of DC vaccines after tumor resection by eliminating immunosuppressive cells. Synergistic effects of DC vaccines and gemcitabine are under investigation in adults and children with sarcoma (NCT01803152). Notably, activation of the NLRP3 inflammasome and the subsequent secretion of IL-1β in MDSCs after Gem and 5-FU treatments may dampen the antitumor efficacy of the two agents.^318^ 5-FU exerted higher antitumor effects when combined with IL-1R antagonists (IL-1Ra) or NLRP3 inflammasome inhibitors in tumor-bearing mice.^319,320^ Further, the IRAFU study (NCT02090101) reported that 5-FU in combination with bevacizumab and anakinra had promising efficiency and good safety profile in metastatic colorectal cancer (mCRC) patients who had undergone chemotherapy and anti-angiogenic therapy.^321^

Notably, chemotherapeutic agents have diverse effects on MDSCs under different situations.^159^ It depends on multiple variables including chemotherapy doses, administration schedules, tumor types and stages, as well as the location and sampling time of MDSCs. For instance, cytotoxic drugs such as cyclophosphamide (CTX) and melphalan can induce MDSCs infiltration through chemotherapy-induced inflammatory responses.^322^ In addition, chemotherapy drugs are not specific to MDSCs but affect all rapidly proliferating cells, including antitumor T cells. Therefore, the net impact of chemotherapy on tumor immunity relies on the counterbalance between immunostimulatory and immunosuppressive effects.^323^ Most chemotherapeutic drugs have the potential to eliminate MDSCs, if administered at the proper time and dose with a proper administration mode in the right tumor model.^324^

#### Sunitinib

Sunitinib, a multitargeted tyrosine kinase inhibitor (TKI) with properties to inhibit angiogenesis and modulate immune dysfunction, has been approved for the front-line therapy of mRCC patients.^325^ Besides, sunitinib induces antitumor activities partially through reducing MDSCs levels.^326,327^ Treatment of mRCC patients with sunitinib significantly reduced the MDSCs levels in the peripheral blood, which was associated with a reversal of Tregs elevation.^328^ However, it was reported that the intratumoral MDSCs in 4T1 mouse mammary carcinoma or human RCC tumor mediated the resistant to sunitinib treatment, and the selective expression of GM-CSF within the TME through STAT5 signaling accounted for this resistence.^329^ Moreover, proangiogenic proteins produced by tumors and MDSCs were also important contributors to MDSCs-mediated anti-angiogenesis resistance.^330^

On the other hand, sunitinib therapy increased the efficacy of stereotactic body radiotherapy (SBRT) in patients with oligo-metastases tumors by reversing MDSCs and Tregs-mediated immunosuppression.^331^ In addition, many studies have investigated the synergistic effects of sunitinib in combination with several kinds of immunotherapies on enhancing antitumor benefits in tumor-bearing mice.^162,332^ Recently, a pilot study was conducted in eight mRCC patients treated with autologous tumor lysate-loaded DC vaccine plus sunitinib. Analysis showed no vaccination-related severe adverse events. Moreover, tumor lysate-reactive T cell responses were observed in five patients, four of whom showed decreased frequencies of MDSCs.^333^

#### TRAIL-R agonists

TNF-related apoptosis induced ligand-receptors (TRAIL-Rs) are members of the TNF receptor superfamily and include two death receptors, TRAIL-R1 (DR4/CD261) and TRAIL-R2 (DR5/CD262). Binding of TRAIL to DR4 or DR5 can activate apoptotic pathway in tumors or infected cells.^334^ MDSCs in tumor-bearing mice were reported to have lower viability and shorter half-life than neutrophils and monocytes, which could be attributed to ER stress response-dependent upregulation of TRAIL-Rs in MDSCs.^65^ In a phase I trial comprising 16 patients with advanced cancers, TRAIL-R2 agonistic antibody DS-8273a reduced MDSCs levels in the peripheral blood of most patients and decreased tumor-infiltrating MDSCs in 50% of the patients, without affecting the levels of neutrophils, monocytes, and other myeloid and lymphoid cells.^335^ However, another study reported that stimulation of TRAIL-R in cancer cells induced tumor-derived CCL2 production, thus increasing the accumulation of M2-type cells and MDSCs in the TME. Analysis of the RNA sequencing data from a cohort of 489 lung adenocarcinoma patients showed that TRAIL expression was positively correlated with the expression of M2 myeloid cell markers and cytokines, such as CD206 and CCL2.^336^ Therefore, further studies are required to investigate the immunomodulatory roles of TRAIL-Rs on immune cells in tumors.^337^

#### Anti-CD33

CD33 is a therapeutic target on MDSCs across different cancer subtypes in human.^338^ Recently, a fully humanized, Fc-engineered mAb against CD33 known as BI 836858 has been proved to inhibit CD33-mediated signal transduction in myelodysplastic syndrome (MDS) patients.^339^ The findings indicated that BI 836858 depleted MDSCs through Ab-dependent cell-mediated cytotoxicity (ADCC). In addition, the immunotoxin gemtuzumab ozogamicin (a humanized mAb targeting CD33 and is approved for the treatment of CD33-positive AML) was reported to decrease MDSCs levels and reactivate T cell and CAR-T cell effects against multiple cancers in vitro.^338^

### MDSCs-targeting strategies in combination with immunotherapies

Recently, immunotherapy has greatly changed the status of cancer treatment, and numerous immunotherapies have been approved by the FDA, among which immune checkpoint inhibitor is the most promising therapy. Moreover, other types of immunotherapies such as mAbs targeting tumor-associated antigens, cancer vaccines, adoptive immune cells therapies, and unspecific boosting of the immune system with ILs, IFNs, or TLR-ligands are currently under investigation. However, anticancer effects of these treatments are limited. MDSCs play critical roles in immunotherapeutic resistance by dampening the host immune responses against tumors. Consequently, alternative strategies targeting MDSCs combined with active or passive immunotherapies will generate synergistic effects. These combinatory therapies have been explored in tumor-bearing mice, and some are being tested in clinical trials (Table 2).

**Table 2:** Clinical trials evaluating MDSCs-targeting strategies in combination with immunotherapy in cancer.

	Target	Drug name	Combination therapy	Indications	Phase	Last reported status	NCT number
Inhibiting expansion and recruitment	GM-CSF	Sargramostim	Ipilimumab	Melanoma	II	Active, not recruiting	NCT01134614
	CSF-1R	Cabiralizumab	Nivolumab	Solid tumors	I	Active, not recruiting	NCT02526017
	VEGF	Bevacizumab, Entinostat	Atezolizumab	Metastatic cancer, Renal cancer	I/II	Recruiting	NCT03024437
	VEGF	Bevacizumab, IPI-549	Atezolizumab	Breast cancer, Renal cell carcinoma	II	Recruiting	NCT03961698
	EGFR	Cetuximab	Ipilimumab	Head and neck cancer	I	Active, not recruiting	NCT01935921
	VEGFR	Cabozantinib, S-malate	Nivolumab+Ipilimumab	Thyroid cancer	II	Recruiting	NCT03914300
	VEGFR	Regorafenib	Nivolumab	Hepatocellular carcinoma	I/II	Recruiting	NCT04170556
	IL-1β	Canakinumab	Spartalizumab	Renal cell carcinoma	I	Recruiting	NCT04028245
	IL-8	BMS-986253	Nivolumab	Cancer	I/II	Active, not recruiting	NCT03400332
	CXCR1/2	Navarixin	Pembrolizumab	Solid tumors	II	Active, not recruiting	NCT03473925
	CXCR1/2	SX-682	Nivolumab	Colorectal cancer	I/II	Recruiting	NCT04599140
	CXCR1/2	SX-682	Nivolumab	Pancreatic cancer	I	Recruiting	NCT04477343
	CXCR1/2	SX-682	Pembrolizumab	Melanoma	I	Recruiting	NCT03161431
	CCR2/CCR5	BMS-813160	Nivolumab	Pancreatic ductal denocarcinoma	I/II	Recruiting	NCT03767582
	CCR2/CCR5	BMS-813160	Nivolumab	Pancreatic ductal denocarcinoma	I/II	Recruiting	NCT03496662
	CCR2/CCR5	BMS-813160	Nivolumab	Colorectal cancer, Pancreatic cancer	I/II	Recruiting	NCT03184870
	CCR2/CCR5	BMS-813160, BMS-986253	Nivolumab	NSCLC, Hepatocellular carcinoma	II	Recruiting	NCT04123379
	CCR5	Vicriviroc	Pembrolizumab	Colorectal neoplasms	II	Active, not recruiting	NCT03631407
	PI3K	IPI-549	Nivolumab	Cancer	I	Active, not recruiting	NCT02637531
Promoting differentiation	STAT3	AZD9150	MEDI4736	Malignant neoplasm of digestive, respiratory or intrathoracic organ	II	Recruiting	NCT02983578
	STAT3	AZD9150, AZD5069	MEDI4736, Tremelimumab	Advanced solid tumors	I/II	Active, not recruiting	NCT02499328
	RAR/RXR	ATRA	Ipilimumab	Melanoma	II	Active, not recruiting	NCT02403778
	RAR/RXR	ATRA	Pembrolizumab	Melanoma	I/II	Recruiting	NCT03200847
	RAR/RXR	ATRA, Cyclophosphamide	Vaccine	Lung cancer	II	Completed	NCT00601796
	RAR/RXR	ATRA, Paclitaxel	Ad.p53-DC vaccine	SCLC	II	Completed	NCT00617409
	TLR3	Poly ICLC	IMA 950	CNS tumor, Adult	II	Completed	NCT01920191
	TLR7	Imiquimod	DC Vaccine	Malignant glioma, Glioblastoma	I	Active, not recruiting	NCT01808820
	TLR9	CMP-001	Nivolumab	Melanoma, Lymph node cancer	II	Recruiting	NCT03618641
	TLR9	CpG	Nivolumab	Pancreatic cancer	I	Recruiting	NCT04612530
Inhibiting function	COX	Acetylsalicylic acid	Pembrolizumab	Head and neck cancer	I	Recruiting	NCT03245489
	COX-2	Celecoxib	DC Vaccine	Ovarian cancer	I/II	Recruiting	NCT02432378
	PDE5	Tadalafil	Cancer Vaccine	Head and neck carcinoma	I/II	Active, not recruiting	NCT02544880
	PDE5	Tadalafil	Telomerase Vaccine	Pancreatic adenocarcinoma	I	Completed	NCT01342224
	NRF2	Omaveloxolone	Ipilimumab, Nivolumab	Melanoma	I/II	Completed	NCT02259231
	HDAC	Entinostat	Nivolumab	Cholangiocarcinoma, Pancreatic cancer	II	Recruiting	NCT03250273
	HDAC	Entinostat	Nivolumab	NSCLC	II	Recruiting	NCT01928576
	HDAC	Entinostat	Nivolumab, Ipilimumab	Breast cancer	I	Active, not recruiting	NCT02453620
Inhibiting metabolism	Liver-X receptor	RGX-104	Nivolumab, Ipilimumab, Pembrolizumab	Malignant neoplasms	I	Recruiting	NCT02922764
		Metformin	Pembrolizumab	Melanoma	I	Recruiting	NCT03311308
	IDO	Epacadostat	Pembrolizumab	Melanoma	III	Completed	NCT02752074
	IDO	BMS-986205	Nivolumab	Glioblastoma	I	Recruiting	NCT04047706
	CD73	Oleclumab	Durvalumab	Pancreatic ductal, Adenocarcinoma, NSCLC, Head and neck carcinoma	II	Not yet recruiting	NCT04262388
	CD73	Oleclumab	Durvalumab	NSCLC, Renal cell carcinoma	II	Not yet recruiting	NCT04262375
	CD73	LY3475070	Pembrolizumab	Advanced cancer	I	Recruiting	NCT04148937
	CD73	MEDI9447	MEDI4736	Triple negative breast cancer	I/II	Recruiting	NCT03616886
	CD73	MEDI9447	Durvalumab, Tremelilumab, MEDI 0562	Ovarian cancer	I	Recruiting	NCT03267589
	CD73	MEDI9447	MEDI4736	Solid tumors	I	Completed	NCT02503774
	CD73	AK119	AK104	Solid tumors	I	Not yet recruiting	NCT04572152
	CD73	Oleclumab	Durvalumab	Sarcoma	II	Recruiting	NCT04668300
	CD73/A2AR	CPI-006 ciforadenant	Pembrolizumab	Cancer	I	Recruiting	NCT03454451
Depleting MDSCs		Gemcitabine	Nivolumab	NSCLC	IV	Not yet recruiting	NCT04331626
		Gemcitabine	Modified Vaccine	Ovarian cancer	I	Completed	NCT02275039
		Gemcitabine	DC Vaccine	Breast cancer	I	Completed	NCT02479230
		Gemcitabine	DC Vaccine	Sarcoma	I	Active, not recruiting	NCT01803152
		Capecitabine	Avelumab	Colon rectal cancer	II	Recruiting	NCT03854799
		Capecitabine, Cisplatin	Rituximab	Head and neck carcinoma	I	Completed	NCT04361409
		Cyclophosphamide	iNKT cells, hrIL-2	Hepatocellular carcinoma	II/III	Recruiting	NCT04011033
		Cyclophosphamide	Modified T cells	Leukemia	I	Completed	NCT01416974
		Cyclophosphamide	IMA970A plus CV8102	Hepatocellular carcinoma	I/II	Completed	NCT03203005
		Cyclophosphamide	Tecemotide	Rectal cancer	II	Completed	NCT01507103
		Cyclophosphamide, Fludarabine	Peripheral blood transplant	Hematological malignancy	III	Recruiting	NCT03480360
		Cyclophosphamide, Fludarabine	GD2-CAR-expressing Autologous T-lymphocytes	Neuroblastoma osteosarcoma	I	Not yet recruiting	NCT04539366
		Cyclophosphamide, Curcumin, Aspirin, Lansoprazole	Pembrolizumab	Cervical cancer, Endometrial cancer, Uterine cancer	II	Recruiting	NCT03192059
		Fluorouracil, Mitomycin, Cisplatin	Avelumab	Bladder cancer	II	Active, not recruiting	NCT03617913
		Docetaxel	DC Vaccine	Prostatic neoplasms	II	Completed	NCT01446731
		Doxorubicin, Cyclophosphamide, Paclitaxel, Carboplatin, Decitabine	Pembrolizumab	Breast cancer	II	Recruiting	NCT02957968
		Fluorouracil, Gemcitabine, Irinotecan, Oxaliplatin, Paclitaxel	Aldesleukin	Pancreatic cancer	I/II	Active, not recruiting	NCT02620865
		Vinorelbine	Atezolizumab	NSCLC	II	Recruiting	NCT03801304
	MEK1	Cobimetinib	Atezolizumab	Gallbladder carcinoma, Cholangiocarcinoma	II	Active, not recruiting	NCT03201458
	AKT	Ipatasertib	Atezolizumab	Solid tumor	I/II	Active, not recruiting	NCT03673787
	BTK	Ibrutinib	Nivolumab	Metastatic malignant solid neoplasm	I	Active, not recruiting	NCT03525925
	MET/VEGFR1/VEGFR/ROS1/RET/AXL/NTRK/KIT	Cabozantinib	Ipilimumab, Nivolumab	Large cell neuroendocrine carcinoma, Neuroendocrine carcinoma, Small cell carcinoma	II	Recruiting	NCT04079712
Immunotherapy		Nivolumab	Ipilimumab	Renal cell cancer	I	Recruiting	NCT03829111
		Ipilimumab	Nivolumab	Renal cell carcinoma	II	Active, not recruiting	NCT02917772
		Ipilimumab	Nivolumab	Melanoma	II	Active, not recruiting	NCT02374242
		Ipilimumab	Nivolumab	Acute myeloid leukemia	I	Recruiting	NCT02846376
Other therapies		Modified vaccinia virus ankara vaccine expressing p53	Pembrolizumab	Ovarian cancer	II	Recruiting	NCT03113487

## Discussion and future perspectives

Currently, MDSCs remain extremely heterogenic populations that are blocked at different differentiation stages and are located in various organs of tumor-bearing individuals. Factors modulating the expansion, activation, and differentiation of MDSCs are closely connected and even overlapped. Notably, plasticity and heterogeneity represent two major challenges in MDSCs research. However, to date, the specific markers for MDSCs are not consensually defined. It requires further work, potentially with the use of high-throughput proteomics and genomics technologies, to clarify and maintain synchrony in the nomenclature and characterization of MDSCs in cancer. Besides, only a few studies have explored tumor-infiltrating MDSCs, probably due to the challenges in isolating MDSCs which are intricately attached to tumor cells. Furthermore, although most of the current studies are focusing on the total MDSCs populations, in fact, the regulatory mechanisms of different MDSC subtypes are likely to be distinct. Therefore, identification and illustration of the unique regulatory and functional mediators of MDSCs will ensure more accurate targeting of specific MDSC subtypes.

Owing to the versatility of MDSCs and the complexity of tumor microenvironment, the inhibitory mechanisms of MDSCs are not likely to function simultaneously, making it challenging to determine the predominant target against MDSCs. In addition, the phenotypic similarity between MDSCs and normal myeloid cells makes it challenging to selectively target MDSCs. Therefore, the design of clinical trials targeting MDSCs in cancer patients should consider several factors such as the tumor sites and stages, the tumor pathological types, the antitumor therapies (especially treatments affecting myeloid hematopoiesis), and the intervals between treatment and blood sampling.

In the last decade, various drugs and compounds have been reported to directly or indirectly inhibit MDSCs in cancer, among which some have been approved by the FDA, some are undergoing clinical trials, and others are investigated in preclinical models. However, MDSCs generation, expansion, recruitment, activation, and immunosuppression involve complex mechanisms, hence it seems impossible for a single approach to control or delete MDSCs and in turn induce powerful antitumor effects. Therefore, the combination of MDSCs-targeting treatments and other anticancer therapies should be the preferred strategy. Nevertheless, when used in combination, dosage, scheduling, and treatment succession should be carefully determined.
